# Bacterial Cyclopropane Fatty Acid Synthase mRNA Is Targeted by Activating and Repressing Small RNAs

**DOI:** 10.1128/JB.00461-19

**Published:** 2019-09-06

**Authors:** Colleen M. Bianco, Kathrin S. Fröhlich, Carin K. Vanderpool

**Affiliations:** a Department of Microbiology, University of Illinois, Urbana, Illinois, USA; b Microbiology, Ludwig Maximilians University, Munich, Germany; Princeton University

**Keywords:** cyclopropane fatty acid synthase, Hfq, RNase E, lipid modification, posttranscriptional regulation

## Abstract

Altering membrane protein and lipid composition is an important strategy for maintaining membrane integrity during environmental stress. Many bacterial small RNAs (sRNAs) control membrane protein production, but sRNA-mediated regulation of membrane fatty acid composition is less well understood. The sRNA RydC was previously shown to stabilize *cfa* (cyclopropane fatty acid synthase) mRNA, resulting in higher levels of cyclopropane fatty acids in the cell membrane. Here, we report that additional sRNAs, ArrS and CpxQ, also directly regulate *cfa* posttranscriptionally. RydC and ArrS act through masking an RNase E cleavage site in the *cfa* mRNA 5′ untranslated region (UTR), and both sRNAs posttranscriptionally activate *cfa*. In contrast, CpxQ binds to a different site in the *cfa* mRNA 5′ UTR and represses *cfa* expression. Alteration of membrane lipid composition is a key mechanism for bacteria to survive low-pH environments, and we show that *cfa* translation increases in an sRNA-dependent manner when cells are subjected to mild acid stress. This work suggests an important role for sRNAs in the acid stress response through regulation of *cfa* mRNA.

**IMPORTANCE** Small RNAs (sRNAs) in bacteria are abundant and play important roles in posttranscriptional regulation of gene expression, particularly under stress conditions. Some mRNAs are targets for regulation by multiple sRNAs, each responding to different environmental signals. Uncovering the regulatory mechanisms governing sRNA-mRNA interactions and the relevant conditions for these interactions is an ongoing challenge. In this study, we discovered that multiple sRNAs control membrane lipid composition by regulating stability of a single mRNA target. The sRNA-dependent regulation occurred in response to changing pH and was important for cell viability under acid stress conditions. This work reveals yet another aspect of bacterial physiology controlled at the posttranscriptional level by sRNA regulators.

## Abstract (precis)

Small RNAs (sRNAs) in bacteria are abundant and play important roles in posttranscriptional regulation of gene expression, particularly under stress conditions. Some mRNAs are targets for regulation by multiple sRNAs, each responding to different environmental signals. Uncovering the regulatory mechanisms governing sRNA-mRNA interactions and the relevant conditions for these interactions is an ongoing challenge. In this study, we discovered that multiple sRNAs control membrane lipid composition by regulating stability of a single mRNA target. The sRNA-dependent regulation occurred in response to changing pH and was important for cell viability under acid stress conditions. This work reveals yet another aspect of bacterial physiology controlled at the posttranscriptional level by sRNA regulators.

## INTRODUCTION

Bacteria modify the biophysical properties of their membranes to adapt to changing environmental conditions, such as pH, temperature, and pressure fluctuations (1). Membrane properties can be altered by changing the type or abundance of proteins embedded in the membrane or by modifying the relative proportions of different types of phospholipids. Membrane fluidity is a crucial biophysical property, as it affects membrane-associated functions, such as permeability to solutes, solute transport, and protein-protein interactions. The length and saturation of the acyl chains in the phospholipids determine the fluidity of the membrane, and bacteria adjust the ratio of saturated to unsaturated fatty acids (UFAs) to adapt to their environment. For example, bacteria can increase resistance to toxic compounds, such as antimicrobial peptides, by increasing the production of saturated fatty acids (1), which will form a tightly packed and less fluid membrane than a membrane rich in unsaturated fatty acids.

While *de novo* production of fatty acids is an important adaptation mechanism, bacteria may encounter abrupt environmental changes that require rapid modification of the fatty acids already incorporated in the membrane. One postsynthetic modification is the conversion of a preexisting UFA to a cyclopropane fatty acid (CFA) by the enzyme cyclopropane fatty acid synthase (encoded by *cfa*). CFAs are formed by the addition of a methylene group, derived from *S*-adenosylmethionine, across the double bond of a UFA incorporated in a phospholipid (2, 3). CFAs occur in the phospholipids of many species of bacteria, but although widely studied, their physiological function remains unclear. One hypothesis is that the formation of CFAs may reduce membrane fluidity and permeability (2), including the permeability to protons (4). Indeed, Escherichia coli *cfa* mutants are sensitive to acid shock, a rapid shift from pH 7 to pH 3 (5, 6). In addition, pathogenic E. coli strains contain more CFAs in their phospholipids and are more resistant to acid than nonpathogenic E. coli strains (5). The formation of CFAs has been further linked to bacterial virulence, as inactivation of a *cfa* homologue that introduces a cyclopropane ring in the major cell envelope component (α-mycolates) of Mycobacterium tuberculosis leaves the bacterium unable to establish a persistent infection (7).

**FIG 1:**
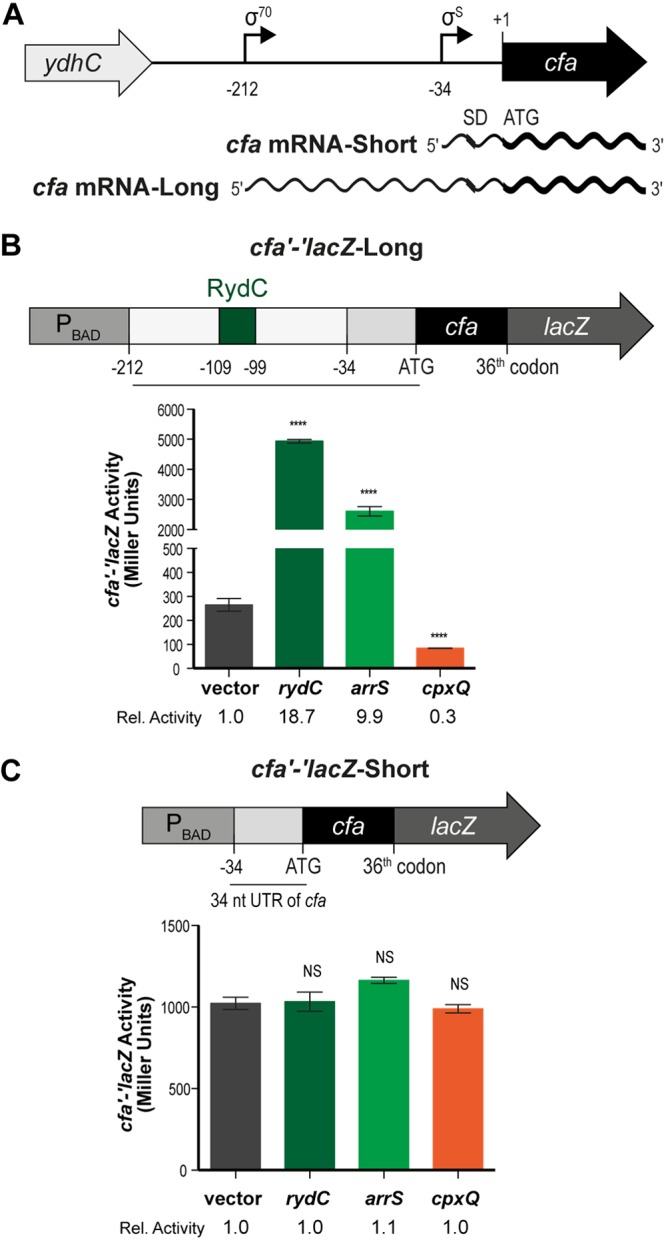
*cfa* expression is controlled by multiple sRNAs. (A) *cfa* has two promoters. Transcription from the distal promoter is σ^70^ dependent and yields a longer *cfa* transcript with a 212-nt 5′ UTR. Transcription from the proximal promoter is controlled by σ^s^ and produces a shorter *cfa* transcript with a 34-nt 5′ UTR. (B) A *cfa* translational fusion to *lacZ* (controlled by the P_BAD_ promoter) was constructed. The *cfa'-'lacZ*-Long fusion is from the distal σ^70^-dependent promoter, which contains a 212-nt 5′ UTR that includes the RydC binding site (square labeled RydC). *cfa'-'lacZ*-Long expression strain carrying an empty or a P*_lac_*-sRNA plasmid was grown in TB medium with 0.002% l-arabinose to early exponential phase, and then sRNA expression was induced with 0.1 mM IPTG. Samples were harvested 60 min later and assayed for β-galactosidase activity of the reporter fusion. The error bars are standard deviations of the results of three independent experiments, and the statistical significance was determined using two-tailed Student's *t* test. ****, *P* < 0.0001. Student's *t* test was performed by comparing the fusion activity in response to the expression of each sRNA to the fusion activity in the vector control. (C) A *cfa'-'lacZ*-Short fusion was constructed; it contains only the σ^s^-dependent promoter and consequently not the predicted sRNA binding sites. Regulation of *cfa'-'lacZ*-Short by each sRNA was determined as described for panel B. NS, not significant.

Production of CFAs is regulated at multiple levels. In E. coli and *Salmonella*, *cfa* transcription is driven by two promoters (8, 9). The distal promoter is σ^70^ dependent and yields a long *cfa* transcript with a 212-nucleotide (nt) 5′ untranslated region (UTR). The proximal promoter is controlled by the general stress response σ factor (σ^s^) encoded by *rpoS* and produces a short *cfa* transcript with a 34-nt 5′ UTR (Fig. 1A). The σ^70^-dependent promoter is functional throughout growth, whereas transcription from the σ^s^ promoter occurs only during stationary phase.

Posttranscriptional regulation by small RNAs (sRNAs) constitutes a major level of gene expression control in bacteria. In many cases, a ubiquitous RNA chaperone, Hfq, is required for sRNA stability and to mediate base-pairing interactions between an sRNA and its cognate target mRNAs. A recent study applied a methodology based on ligation of Hfq-associated RNAs and sequencing of chimeric fragments to globally map RNA interactions *in vivo* (10). This approach (termed RIL-seq) uncovered potential *cfa* mRNA interactions with multiple sRNAs in E. coli: ArrS, CpxQ, and RydC. RydC was previously reported to stabilize *cfa* mRNA, which in turn increases levels of CFA synthase and results in proportionally more CFAs in membrane lipids (11). RydC, a 64-nt-long sRNA, folds in a characteristic pseudoknot structure which exposes a stretch of highly conserved nucleotides at the very 5′ end of the RNA. Binding of RydC with the long isoform *cfa* mRNA masks a recognition site for the major endonuclease RNase E and inhibits transcript degradation. In addition, RydC has been reported to regulate *yejABEF* (12), *csgD* (13), *pheA*, and *trpE* (14) mRNAs, although the conditions and signals stimulating RydC production and the role of RydC in bacterial physiology remain unknown. In contrast, ArrS and CpxQ sRNAs were previously linked to cell envelope or acid stress responses. Production of the CpxQ sRNA depends on the Cpx two-component system that responds to cell envelope stress (15). CpxQ is processed by RNase E from the 3′ UTR of *cpxP* mRNA, which encodes a chaperone of the Cpx system (15). Together with Hfq, CpxQ represses translation of multiple targets, many of which encode inner membrane proteins (15, 16). ArrS is an antisense sRNA that is encoded upstream of *gadE* (encoding the major acid resistance transcription factor) and is complementary to the 5′ UTR of the longest of three *gadE* transcripts (17). ArrS expression is induced by low pH through σ^s^ and GadE, and overexpression of ArrS increases survival of cells exposed to acidic pH (18).

In this study, we verified direct base pairing-dependent regulation of *cfa* mRNA by two additional sRNAs, ArrS and CpxQ, demonstrating that the long isoform of *cfa* mRNA is a target for positive and negative posttranscriptional regulation by sRNAs. Our results indicate that activating sRNAs RydC and ArrS both utilize overlapping binding sites in the long 5′ UTR of *cfa* mRNA and that sRNA binding to these sites protects *cfa* mRNA from cleavage by RNase E. This study also describes the first phenotype associated with sRNA-dependent regulation of *cfa.* RydC-dependent activation increases *cfa* expression at acidic pH, and *rydC* mutant cells exhibit an acid-sensitive phenotype. Unlike RydC and ArrS, the sRNA CpxQ represses *cfa* expression posttranscriptionally. CpxQ binds at a distinct site on *cfa* mRNA, upstream from the site bound by activating sRNAs. Our results suggest that Hfq and CpxQ binding to the 5′ region of *cfa* mRNA modulates susceptibility to cleavage by RNase E at the downstream site. All together, our work implicates activating and repressing sRNAs in the control of membrane fatty acid composition via regulation of *cfa* mRNA stability.

## RESULTS

### Multiple sRNAs regulate CFA synthase mRNA in an isoform-specific manner.

Transcription of *cfa* is controlled by two promoters that yield mRNAs with a 212-nt 5′ UTR (distal σ^70^-dependent promoter) or a 34-nt 5′ UTR (σ^S^-dependent promoter) (8) (Fig. 1A). Only the long mRNA isoform is subject to posttranscriptional regulation by the sRNA RydC, which stabilizes the transcript by base-pairing at an RNase E recognition site within the 5′ UTR (11). An experimental study based on the ligation of Hfq-associated RNAs (RIL-seq [10]) identified RydC cross-linked and ligated to *cfa* mRNA and, in addition, revealed potential interactions of the *cfa* transcript with ArrS and CpxQ sRNAs. To test whether ArrS and CpxQ sRNAs alter *cfa* expression posttranscriptionally *in vivo*, we constructed two translational *cfa'-'lacZ* fusions under the control of the arabinose-inducible P_BAD_ promoter (14). One fusion contains the 212-nt 5′ UTR and the first 36 codons of *cfa* (*cfa'-'lacZ*-Long) (Fig. 1B), while the second has the short 34-nt 5′ UTR and the first 36 codons of *cfa* (*cfa'-'lacZ*-Short) (Fig. 1C). We ectopically expressed each sRNA from a plasmid (P_lac_ promoter) and determined the effect on the two reporter fusions. RydC and ArrS increased *cfa'-'lacZ*-Long activity 18- and 10-fold, respectively, while CpxQ repressed the long fusion ∼3-fold (Fig. 1B). None of these sRNAs had an effect on *cfa'-'lacZ*-Short activity (Fig. 1C). These results indicate that the long isoform of *cfa* mRNA can be posttranscriptionally activated or repressed by multiple sRNAs, while the short isoform is not affected by these regulatory molecules.

RIL-seq (10) also identified potential interactions of the *cfa* mRNA with OxyS, GadF, and GcvB sRNAs. We tested *cfa'-'lacZ*-Long and *cfa'-'lacZ*-Short for regulation by OxyS, GadF, and GcvB (see Fig. S1 in the supplemental material). Expression of OxyS increased *cfa'-'lacZ*-Long activity ∼2-fold, and GadF increased *cfa'-'lacZ*-Long activity very modestly (∼1.2-fold), while expression of GcvB slightly decreased *cfa'-'lacZ*-Long activity (Fig. S1A). None of these sRNAs significantly affected *cfa'-'lacZ*-Short activity (Fig. S1B). To determine if the minor regulation of *cfa'-'lacZ*-Long by OxyS, GadF, and GcvB is mediated by direct sRNA-mRNA base-pairing interactions, we used IntaRNA (19) to predict sRNA-mRNA binding interactions (Table S1), followed by *in vitro* footprinting (Fig. S1C). For the footprinting experiments, we digested an ∼140-nt fragment of *cfa* mRNA (−212 to −72 relative to the start codon) in the presence of Hfq and each of the three candidate sRNAs with RNase T_1_ or lead(II) acetate (Fig. S1C). We did not detect binding of GadF (Fig. S1C, lanes 5 and 9) or OxyS (Fig. S1C, lanes 6 and 10) to *cfa* mRNA. We did detect a weak GcvB-*cfa* mRNA interaction (Fig. S1C, lane 7), consistent with the IntaRNA prediction (Table S1). OxyS is expressed under oxidative stress and represses *rpoS* expression by an unknown mechanism (20, 21). GcvB mutants are acid sensitive, and this sensitivity may be due to reduced RpoS levels; however, how GcvB activates RpoS expression is not known (22). Due to the potential regulation of RpoS by OxyS and GcvB (which could indirectly regulate *cfa'-'lacZ*-Long by modulating expression from the σ^S^-dependent promoter), the minor effect of these sRNAs on *cfa* regulation, and little or no evidence of direct interactions *in vitro*, we did not further characterize regulation of *cfa* mRNA by OxyS, GadF, or GcvB.

### Activating and repressing sRNAs bind distinct sites on *cfa* mRNA.

**FIG 2:**
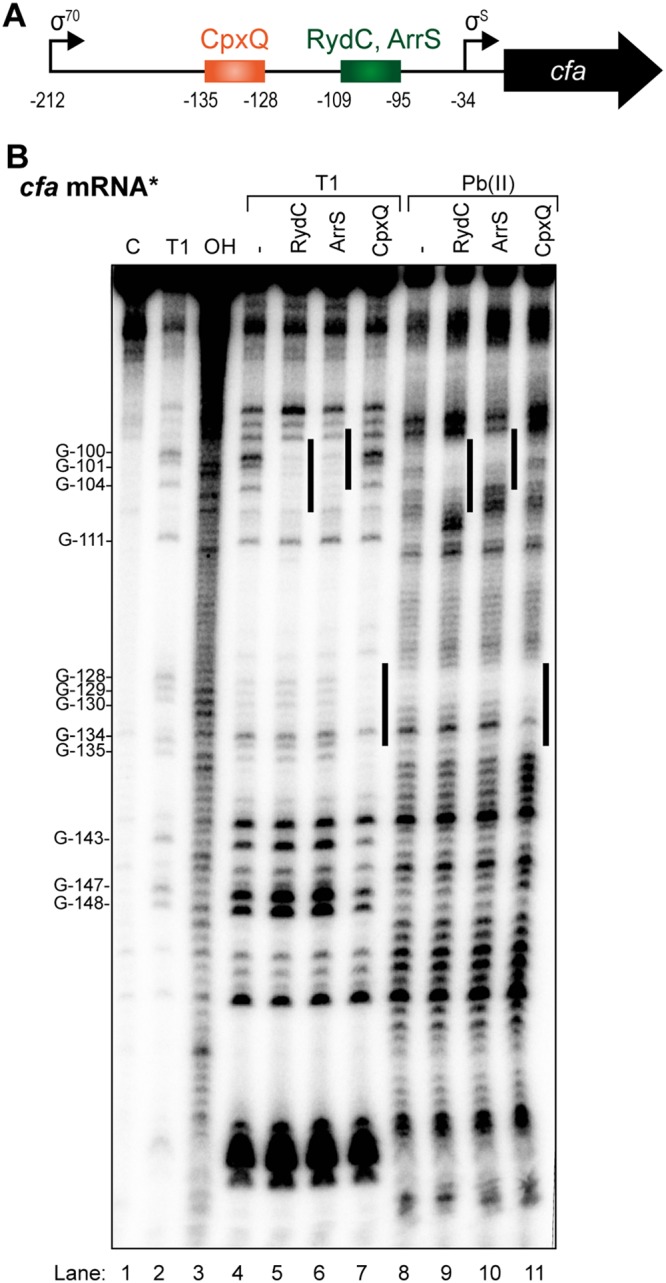
Activating and repressing sRNAs bind different sites on *cfa* mRNA. (A) Predicted base-pairing sites of sRNAs on *cfa* mRNA. (B) *In vitro* structure probing using 5′-end-labeled *cfa* mRNA with RNase T_1_ (lanes 4 to 7) and lead(II) acetate (lanes 8 to 11) in the presence of Hfq (20 nM) and each sRNA (200 nM). RNase T_1_ and alkaline ladders of *cfa* mRNA were used to map cleaved fragments. The positions of G residues are indicated relative to the translational start site. Each sRNA binding site is marked with a line to the right of the lane.

RydC, ArrS, and CpxQ all regulated the *cfa* translational fusion containing the *cfa* 212-nt 5′ UTR but not the *cfa* translational fusion containing only the short 34-nt 5′ UTR (Fig. 1B and C). RydC has previously been shown to base pair at an RNase E recognition site located within this region (11). We used IntaRNA (19) to predict base-pairing interactions and found that ArrS is predicted to bind to a site in the *cfa* mRNA 5′ UTR overlapping the RydC binding site, while CpxQ is predicted to bind to a site further upstream (Fig. 2A; Table S1). To genetically test these base-pairing interactions, we first constructed a *cfa'-'lacZ* fusion with a 133-nt deletion from the beginning of the 212-nt 5′ UTR, which contains predicted binding sites for RydC, ArrS, and CpxQ (*cfa'-'lacZ*-ΔsRNABS) (Fig. S2A and B) as well as the RNase E cleavage site protected by RydC. Notably, *cfa'-'lacZ*-ΔsRNABS had higher basal activity than *cfa'-'lacZ*-Long (Fig. 1B; Fig. S2B), likely because the *cfa'-'lacZ*-ΔsRNABS fusion lacks the known RNase E cleavage site, leading to increased stability of the *cfa'-'lacZ*-ΔsRNABS transcript. When each individual sRNA was ectopically expressed from a plasmid, none of the sRNAs affected *cfa'-'lacZ*-ΔsRNABS activity (Fig. S2B), indicating that each sRNA requires sequences upstream of the −79 nt position for regulation of *cfa*. To map where each sRNA binds *cfa* mRNA (Fig. 2A), we performed structure probing experiments (Fig. 2B). We incubated an ∼140-nt fragment of *cfa* mRNA (−212 to −72 relative to the start codon) with Hfq and each of the three sRNAs and digested them with RNase T_1_ or lead(II) acetate (Fig. 2B). In experiments without added Hfq, we did not see sRNA-mediated protection (data not shown). In the presence of Hfq, we did not see any Hfq-specific protection, but we did observe binding of RydC (Fig. 2B, lanes 5 and 9) and ArrS (lanes 6 and 10) to overlapping sites and binding of CpxQ (lanes 7 and 11) at a distinct upstream site. The results of the *in vitro* structure probing experiment were in line with computational predictions of base-pairing sites (Table S1), suggesting that the two activating sRNAs, ArrS and RydC, shared a common binding site, whereas CpxQ employed a distinct site to repress *cfa* mRNA.

**FIG 3:**
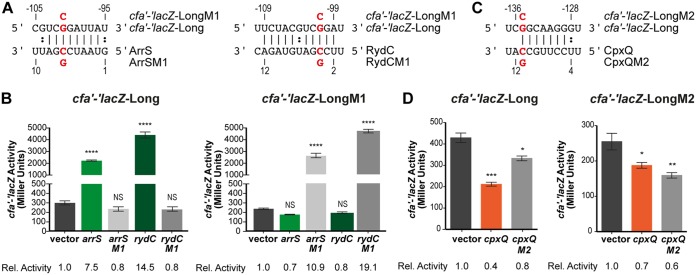
Activating and repressing sRNAs bind different sites on *cfa* mRNA. (A) Predicted base pairing between *cfa* mRNA and ArrS or RydC sRNA. The red nucleotides were mutated to test base pairing. (B) (Left) Mutant sRNAs (ArrSM1, RydCM1) and WT sRNAs were tested for activation of *cfa'-'lacZ*-Long as described in the legend for Fig. 1B. (Right) One point mutation (G101C) was made in *cfa'-'lacZ*-Long (called *cfa'-'lacZ*-LongM1). Mutant sRNAs (ArrSM1, RydCM1) and WT sRNAs were tested for activation of *cfa'-'lacZ*-LongM1 as described in the legend for Fig. 1B. (C) Base pairing between *cfa* mRNA and CpxQ. The red nucleotides were mutated to test base pairing. (D) (Left) Mutant CpxQM2 and WT CpxQ were tested for activation of *cfa'-'lacZ*-Long as described in the legend for Fig. 1B. (Right) One point mutation (G135C) was made in *cfa'-'lacZ*-Long (called *cfa'-'lacZ*-LongM2). Mutant CpxQM2 and WT CpxQ were tested for activation of *cfa'-'lacZ*-LongM2 as described in the legend for Fig. 1B. *, *P* < 0.05; **, *P* < 0.005; ***, *P* < 0.0005; ****, *P* < 0.0001; NS, not significant.

Genetic analyses were conducted to confirm direct sRNA-mRNA base-pairing interactions. We first introduced point mutations in the activating sRNAs, ArrS and RydC (C7G for ArrSM1 and C4G for RydCM1) (Fig. 3A), that disrupted base pairing with *cfa* mRNA. The mutant sRNAs, ArrSM1 and RydCM1, could no longer activate *cfa'-'lacZ*-Long (Fig. 3B, left panel). A compensatory mutation (G101C) in the *cfa* reporter fusion (*cfa'-'lacZ*-LongM1) restored base-pairing interactions with mutant sRNAs (Fig. 3A and B, right panel). Wild-type ArrS and RydC could not activate the mutant *cfa'-'lacZ*-LongM1 fusion (Fig. 3B, right panel). In contrast, RydCM1 and ArrSM1 activated the *cfa'-'lacZ*-LongM1 fusion, with fold activation restored to levels similar to that of the wild-type (WT) sRNA-mRNA pairs (Fig. 3B, right panel).

To characterize CpxQ-mediated regulation of *cfa* mRNA, we introduced a point mutation in CpxQ to disrupt base pairing to *cfa* mRNA (C11G; called CpxQM2) (Fig. 3C). CpxQM2 lost most of the repression activity for *cfa'-'lacZ*-Long (Fig. 3D, left panel). The compensatory mutation in the *cfa* fusion (G135C; called *cfa'-'lacZ*-LongM2), which disrupted base pairing with wild-type CpxQ, correspondingly attenuated the repression by wild-type CpxQ (Fig. 3D, right panel). The mutant pair, CpxQM2 with *cfa'-'lacZ*-LongM2, showed restored regulation in comparison to the mismatched pairs but not back to wild-type levels (Fig. 3D, right panel). Nevertheless, the loss of regulation caused by individual mutations (Fig. 3D) and the clear CpxQ footprint on *cfa* mRNA (Fig. 2B) strongly suggest that CpxQ binds at a site on *cfa* mRNA that is substantially upstream of the binding site of the activating sRNAs.

### Activating and repressing sRNAs modulate RNase E-dependent degradation of *cfa* mRNA.

Previous studies determined that the RydC-*cfa* mRNA base pairing prevents RNase E-mediated decay and stabilizes the mRNA to allow increased translation (11). Because ArrS pairs with the same region of *cfa* mRNA as RydC, ArrS likely regulates *cfa* mRNA stability by the same mechanism. However, the repressive effect of CpxQ must be mediated by a different mechanism. One possibility is that CpxQ negatively regulates *cfa* mRNA by inhibiting the activity of the positively regulating sRNAs. If this were the case, CpxQ should not be able to repress *cfa* reporter fusions in strains lacking RydC. Ectopic production of CpxQ repressed the *cfa'-'lacZ*-Long fusion to similar degrees in wild-type and *ΔcpxQ ΔrydC* strains (Fig. S3), indicating that CpxQ does not act by modulating the activity of RydC on *cfa* mRNA.

**FIG 4:**
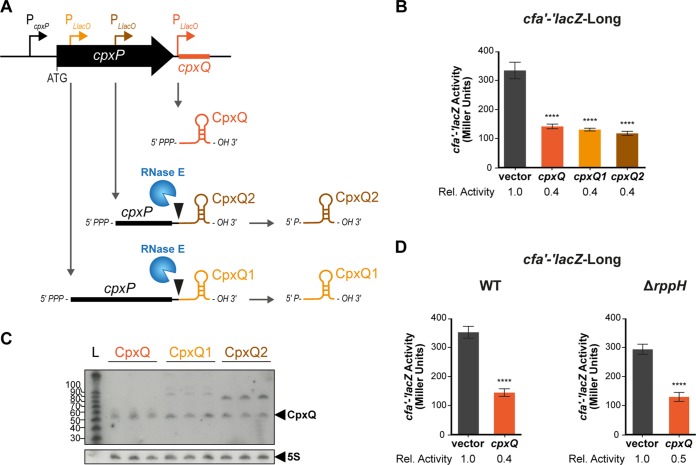
CpxQ does not require a 5′ monophosphate to repress *cfa*. (A) Three CpxQ plasmids were constructed: *cpxQ* encodes CpxQ directly from the *cpxQ* start site, CpxQ1 starts right after the AUG of *cpxP*, and CpxQ2 starts 30 nt upstream of the RNase E cleavage site in the 3′ UTR of *cpxP*. Plasmids were designed so that CpxQ1 and CpxQ2 would be cleaved by RNase E to yield 5′P CpxQ. (B) Regulation of *cfa'-'lacZ*-Long by each sRNA plasmid was tested as described in the legend for Fig. 1B. (C) Expression levels of CpxQ from each plasmid as described for panel A were determined by Northern blot analysis of total RNA samples. 5S served as the loading control. (D) Regulation of *cfa'-'lacZ*-Long by CpxQ was tested in a WT or Δ*rppH* background as described in the legend for Fig. 1B. ****, *P* < 0.0001.

CpxQ represses other targets by base pairing near the Shine-Dalgarno sequence to prevent ribosome binding and directly inhibit translation initiation or by base pairing within the coding region and stimulating mRNA decay by RNase E (15). Since the CpxQ-*cfa* mRNA interaction site within the *cfa* 5′ UTR is far upstream of the Shine-Dalgarno sequence, CpxQ must not repress translation initiation directly. Instead, we hypothesized that CpxQ represses *cfa* by stimulating RNase E-dependent decay. When CpxQ is produced from the native chromosomal locus, it carries a 5′ monophosphate (5′P) because it is a processed product of the *cpxP* mRNA and not a primary transcript. It has been shown that the 5′P ends of sRNAs can stimulate RNase E activity and lead to rapid degradation of the paired mRNA (23). It was demonstrated *in vitro* that RNase E-dependent degradation of another CpxQ target, *nhaB* mRNA, was faster in the presence of 5′P CpxQ than in the presence of 5′PPP CpxQ (15). Additionally, the 5′ end of CpxQ is the seed region used to base pair with both *nhaB* (15) and *cfa* mRNA (Fig. 3C). To determine whether the phosphorylation status of the CpxQ 5′ end impacts its ability to regulate *cfa* mRNA, we designed CpxQ plasmids that would allow for the production of processed 5′P CpxQ (Fig. 4). Three plasmids were used (Fig. 4A). All three plasmids contained DNA regions encompassing mature CpxQ and its ρ-independent terminator. The transcription start site for each plasmid varied (Fig. 4A). The CpxQ plasmid (the same as used in experiments described above) placed the +1 (transcriptional start site) of CpxQ at the native processing site. The CpxQ1 plasmid contained a larger region, beginning in the *cpxP* coding region (just after the *cpxP* start codon). The CpxQ2 plasmid contained a region starting 30 nt upstream of the processing site in the 3′ UTR of *cpxP*. We expected that the CpxQ plasmid would produce CpxQ containing a 5′PPP while the CpxQ1 and CpxQ2 plasmids would produce a *cpxQ* transcript that would be processed by RNase E and yield CpxQ with a 5′P. We tested how each plasmid regulated *cfa'-'lacZ*-Long (Fig. 4B) and found that both CpxQ1 and CpxQ2 repressed *cfa'-'lacZ*-Long activity to the same extent as CpxQ. A Northern blot analysis probing for CpxQ confirmed that CpxQ1 and CpxQ2 were processed to produce the 58-nt CpxQ and that all three plasmids produced the same amount of the 58-nt CpxQ sRNA (Fig. 4C). In addition, deletion of *rppH* (encoding the 5′ pyrophosphatase which converts 5′PPP CpxQ to 5′P CpxQ) in the *cfa'-'lacZ*-Long fusion background did not affect regulation by CpxQ (Fig. 4D). These results suggest that unlike another characterized CpxQ target which requires 5′P for efficient RNase E-mediated mRNA decay (15), the phosphorylation status of the CpxQ 5′ end is not critical for its regulatory activity on *cfa* mRNA.

**FIG 5:**
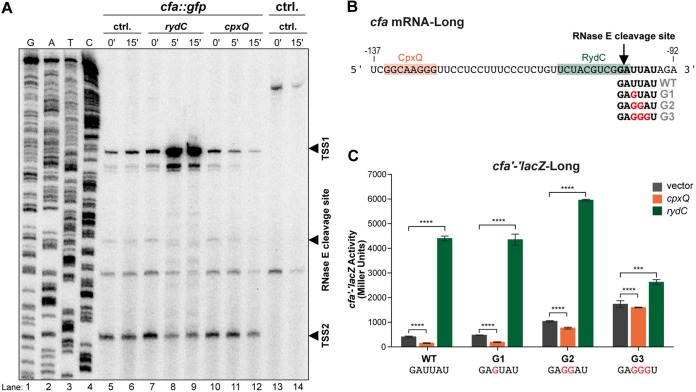
Activating and repressing sRNAs modulate RNase E-dependent degradation of *cfa* mRNA. (A) Primer extension of 5′ UTR of *cfa* mRNA reveals no CpxQ-dependent mRNA cleavage products. RNA samples were withdrawn prior to and 5 or 15 min after arabinose addition to induce the vector control (lanes 5 and 6), RydC (lanes 7 to 9), or CpxQ (lanes 10 to 12). RNA samples were used as the templates for primer extension using a 5′-end-labeled *cfa* primer. Transcripts were identified using *cfa*-specific sequencing ladders (lanes 1 to 4). Transcription start sites 1 (TSS1) and 2 (TSS2) and the known RNase E cleavage site are marked with arrowheads. There is a band present between the RNase E cleavage site product and TSS2. Since this band is also present in the control strain (ctrl, lanes 13 and 14) that lacks *cfa*::*gfp*, we conclude that it is nonspecific and we disregarded it. (B) Multiple mutations of the RNase E recognition site on *cfa* mRNA were made in the *cfa'-'lacZ*-Long fusion background (called G1 to G3). An arrow indicates the RNase E cleavage site. CpxQ and RydC base-pairing locations are indicated. (C) Regulation of *cfa'-'lacZ*-Long (WT) or *cfa'-'lacZ*-LongG1 to *cfa'-'lacZ*-LongG3 by CpxQ and RydC was tested as described in the legend for Fig. 1B. ***, *P* < 0.0005; ****, *P* < 0.0001.

We postulated that CpxQ modulates the stability of *cfa* mRNA by making it more susceptible to degradation. We performed primer extension to profile the mRNA cleavage products generated from the *cfa* 5′ UTR in the absence and presence of RydC and CpxQ (Fig. 5A). Bands representing the two *cfa* mRNA isoforms—transcription start site 1 (TSS1), corresponding to the long isoform (σ^70^ promoter), and TSS2, corresponding to the short isoform (σ^S^ promoter)—were the most dominant signals. Upon RydC induction, we saw a strong accumulation of the longer *cfa* mRNA (TSS1) but no difference in the abundance of the shorter *cfa* transcript (TSS2) (Fig. 5A, lanes 7 to 9). We detected a processed form of the *cfa* mRNA that corresponds to a 5′-GG↓AUU-3′ cleavage site (Fig. 5A, RNase E cleavage site), which is consistent with the cleavage site mapped in *Salmonella* (11). The band corresponding to the mRNA cleavage product disappears when RydC is expressed (Fig. 5A, compare lanes 7 and 9), agreeing with previous work in *Salmonella* demonstrating that RydC binding protects *cfa* mRNA from RNase E-dependent cleavage at this site (11). When CpxQ was induced (Fig. 5A, lanes 10 to 12), we observed a strong reduction of the longer *cfa* mRNA (TSS1) and no effect on the shorter *cfa* (TSS2) transcript. No other bands appeared, suggesting that any CpxQ-dependent mRNA cleavage products were rapidly degraded.

A global study of RNase E cleavage sites (also in *Salmonella*) predicted a 5-nt minimal RNase E consensus sequence of 5′-RN↓WUU-3′(with R as G or A, W as A or U, N as any nucleotide, and ↓ as the cleavage site) (24). In the *Salmonella cfa* mRNA, the sequence surrounding the RNase E cleavage site is GG↓AUAAC. Our primer extension analysis indicates that the major RNase E cleavage site for E. coli *cfa* mRNA is GG↓AUUAU (Fig. S4; Fig. 5A and B). In *Salmonella*, mutation of the U residue 2 nt downstream of the RNase E cleavage site (Fig. S4) significantly reduced the cleavage of *cfa* mRNA by RNase E (24). We used genetics to further validate the putative E. coli RNase E cleavage site and to test whether this site is required for positive regulation of *cfa* by RydC and negative regulation of *cfa* by CpxQ. We first mutated the uridine that is conserved in E. coli and *Salmonella cfa* mRNAs (the E. coli sequence is GG↓AUUAU, with underlining indicating the mutated U residue) to a G (a nonpreferred nucleotide for RNase E recognition) in *cfa'-'lacZ*-Long (G1 mutant) (Fig. 5B and C) and tested for regulation by RydC and CpxQ. Mutation of the conserved U alone in the G1 mutant did not affect the basal level of the fusion compared to the WT (Fig. 5C), suggesting that in E. coli, unlike in *Salmonella*, mutation of this single residue is not sufficient to change the susceptibility of *cfa* mRNA to RNase E cleavage. In addition, CpxQ and RydC regulated the G1 mutant similarly to the wild-type fusion (Fig. 5C).

In some cases, there are uridines at the second and third positions downstream of an RNase E cleavage site and mutation of both U residues is required to fully inhibit cleavage by RNase E (24). To test whether additional residues downstream of the conserved U in the putative E. coli RNase E site are involved in *cfa* mRNA stability and regulation by RydC and CpxQ, we changed two U residues to G’s (G2 mutant) (Fig. 5B and C). These mutations increased the basal activity of *cfa'-'lacZ*-Long ∼2-fold compared to the WT, which is expected if these mutations inhibit cleavage by RNase E (Fig. 5C). The G2 mutation did not impair activation by RydC but substantially impaired CpxQ-mediated repression (Fig. 5C). Mutation of the next residue downstream of the RNase E cleavage site yielded the G3 mutant fusion (Fig. 5B). The basal level of activity was further increased in this fusion, consistent with the model that these mutations impair RNase E cleavage of *cfa* fusion mRNA. Both activation by RydC and repression by CpxQ were strongly impaired in the G3 mutant (Fig. 5C). We used Mfold (25) to compare the predicted structures of wild-type and G3 mutant mRNAs (Fig. S5), and the predicted structures around the CpxQ and RydC binding sites were the same, suggesting that the two sRNAs could still access their binding sites on the G3 mutant mRNA. These results led us to two important conclusions. First, the nucleotide sequence determinants of *cfa* mRNA sensitivity to RNase E are different between E. coli and *Salmonella*. Moreover, the data strongly suggest that the regulation of *cfa* mRNA by both activating and repressing sRNAs involves modulation of cleavage at a single RNase E site adjacent to the activating sRNA binding site but substantially downstream from the repressing sRNA binding site.

### Role of Hfq in sRNA-dependent regulation of *cfa* mRNA.

**FIG 6:**
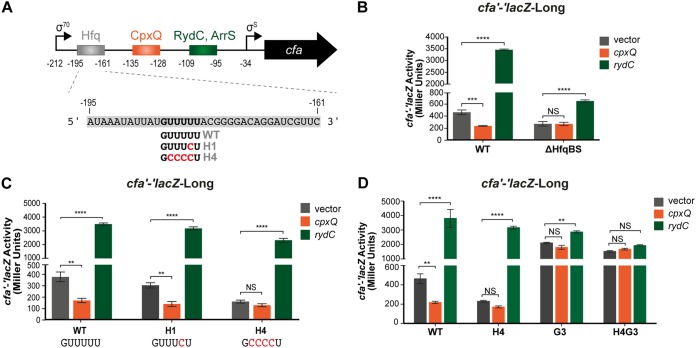
Mutating a putative Hfq binding site alters *cfa* mRNA regulation by CpxQ and RydC. (A) 5′ UTR of the *cfa* gene with a putative Hfq binding site and sRNA base-pairing sites indicated. (B) The first 50 nt of the 212-nt 5′ UTR (−212 to −163), which includes the putative Hfq binding site, was deleted in the *cfa'-'lacZ*-Long fusion (called *cfa'-'lacZ*-ΔHfqBS). Regulation of *cfa'-'lacZ*-Long or *cfa'-'lacZ*-ΔHfqBS by CpxQ and RydC was tested as described in the legend for Fig. 1B. (C) Mutations in the Hfq binding site on *cfa* mRNA (shown in panel A) were made in the *cfa'-'lacZ*-Long fusion background (called *cfa'-'lacZ*-LongH1 and *cfa'-'lacZ*-LongH4). Regulation of WT and mutant fusions by RydC and CpxQ was tested as described in the legend for Fig. 1B. (D) Mutations of either the Hfq binding site on *cfa* mRNA (shown in panel A) (*cfa'-'lacZ*-LongH4), the RNase E recognition site on *cfa* mRNA (shown in Fig. 5B) (*cfa'-'lacZ*-LongG3), or both the Hfq binding site and the RNase E recognition site (*cfa'-'lacZ*-LongH4G3) were made. Regulation of WT and mutant fusions by RydC and CpxQ was tested as described in the legend for Fig. 1B. **, *P* < 0.005; ***, *P* < 0.0005; ****, *P* < 0.0001; NS, not significant.

In a previous study of RydC-mediated regulation of *cfa* mRNA, it was shown that swapping the RydC binding site on *cfa* mRNA for the binding site of another Hfq-dependent sRNA (e.g., RybB or RyhB) reprogrammed the *cfa* mRNA to be activated by the corresponding sRNA (11). However, swapping the RydC binding site with an Hfq-independent sRNA binding site had only a small activating effect (∼2-fold versus 10-fold by RydC) (11). This observation suggested that Hfq plays a key role in sRNA-dependent regulation of *cfa* mRNA stability. An Hfq binding site at the very 5′ end of the UTR of *Salmonella cfa* mRNA (Fig. 6A, −195 to −161) was identified by UV cross-linking and immunoprecipitation (CLIP-seq) (26), but its role in the regulation of *cfa* by RydC has not been studied. To further characterize the role of this Hfq binding site on sRNA-dependent regulation of *cfa* mRNA, we deleted the first 50 nt of the 5′ UTR (−212 to −163) (Fig. 6A and B) (called *cfa'-'lacZ*-ΔHfqBS), which includes the Hfq binding site (−195 to −161). This fusion had lower basal activity than *cfa'-'lacZ*-Long (∼2-fold decrease) (Fig. 6B), suggesting that the Hfq binding site located in the −212 to −163 region of the *cfa* mRNA plays a role in mRNA structure or stability. Regulation of *cfa'-'lacZ*-ΔHfqBS by both activating and repressing sRNAs was strongly impaired, even though the fusion retains both sRNA binding sites (Fig. 6B).

To better understand the importance of the −212 to −163 region and Hfq binding site for sRNA-mediated regulation of *cfa* mRNA, we conducted mutational analysis. CLIP‐seq captured a U-to-C cross-linking‐induced mutation for the *Salmonella* Hfq-*cfa* mRNA interaction, as indicated in the H1 mutant in Fig. 6A (26). The E. coli fusion with the single U-to-C mutation, *cfa'-'lacZ*-LongH1, had a basal level of activity similar to that of the WT and was regulated normally by CpxQ and RydC (Fig. 6C). The sequences of *Salmonella* and E. coli *cfa* mRNAs are not identical in this region (Fig. S4). Notably, there is a run of U residues in this region in E. coli (5′-UGUUUUUAC-3′) but not in *Salmonella* (5′-GGCAUUAA-3′). To test whether the additional U residues in E. coli *cfa* mRNA contribute to regulation by sRNAs, we also made the H4 mutant, with a total of four U-to-C substitutions (Fig. 6A). The H4 mutant had a lower basal level of activity than the WT and the H1 mutant, but at ∼150 U of activity, we expected we should still be able to see repression by CpxQ and activation by RydC if the Hfq binding site was not required for the regulation. What we observed was that CpxQ no longer regulated the *cfa'-'lacZ*-LongH4 fusion, but regulation by RydC remained at near-wild-type levels (Fig. 6C). This result suggests that Hfq binding at this upstream site may be particularly important for CpxQ-mediated repression and less important for RydC-mediated activation of *cfa*.

Basal activity of the *cfa'-'lacZ*-LongH4 fusion was reduced compared to that of the WT and the H1 fusion (Fig. 6C). We reasoned that this reduced activity might be due to the reduced stability of the H4 fusion mRNA and that this could be mediated by increased RNase E-dependent cleavage at the known site (adjacent to the activating sRNA binding site) or by cleavage at other sites. To determine if the reduction in *cfa'-'lacZ*-LongH4 activity was dependent on the known RNase E cleavage site, we combined the H4 (Fig. 6A) and G3 (Fig. 5B) mutations and compared the activities of the single and double mutant fusions. We expected that if the H4 mutation was destabilizing the fusion mRNA by promoting cleavage at a new site, the H4G3 double mutant would have a lower basal level of activity than the G3 mutant. In contrast, if the H4 mutation was destabilizing the fusion mRNA by promoting cleavage at the known site, the H4G3 mutant would have the same basal level of activity as the G3 single mutant. The results of this experiment show that the basal levels of activity of the G3 single and H4G3 double mutant fusions are similar and that both are strongly increased compared to that of the WT and the H4 single mutant fusion (Fig. 6D). Neither the G3 nor the H4G3 fusions are regulated by CpxQ or RydC (Fig. 6D), consistent with the model that both activating and repressing sRNAs modulate *cfa* mRNA stability by affecting cleavage at the same RNase E-sensitive site. Our interpretation of these results is that the H4 mutation affects RNase E cleavage at the site used by both activating and repressing sRNAs.

To further understand the role of Hfq binding in the nt −195 to –161 region in sRNA-mediated regulation of *cfa* mRNA, we examined the 5′ UTR secondary structure. The structure of the 5′ UTR predicted by Mfold (25) suggests that the CpxQ binding site is sequestered in a hairpin, which may make this site inaccessible to CpxQ in the absence of Hfq (Fig. S6A). This predicted structure is consistent with the footprinting assay (Fig. 2B) showing that the CpxQ binding site is not very accessible in comparison to the region just upstream of the binding site. The RydC base-pairing site is less structured (Fig. S6A) and more accessible for RydC binding, again consistent with the footprinting assay showing that the G residues in the RydC binding site are accessible to cleavage by RNase T_1_ (Fig. 2B, lanes 4 and 5). We hypothesize that Hfq binding at the −195 to −161 region would remodel the 5′ UTR to promote CpxQ binding.

A recent study (27) mimicked Hfq binding on a target mRNA by annealing a DNA oligonucleotide complementary to the Hfq binding site and showed that annealing of this DNA oligonucleotide was sufficient to remodel RNA secondary structure, bypassing the requirement for Hfq and allowing sRNA binding. We used the same approach to test our model. We annealed a DNA oligonucleotide complementary to the −195 to −161 Hfq binding region (called oligo H1) (Fig. S6B) with *cfa* mRNA and completed a structure probe in the absence and presence of sRNAs. Importantly, these experiments were performed without added Hfq. In this experiment (Fig. S6B), we could clearly see oligonucleotide-mediated protection of the Hfq binding region (Fig. S6B, compare lanes 4 to 6 with lanes 7 to 9 and lanes 10 to 12 with lanes 13 to 15). In the oligo H1-bound structures (Fig. S6B, lanes 7 to 9 and lanes 13 to 15), we also saw differences in the accessibility of residues just upstream of the CpxQ binding site (G residues at positions −143, −147, and −148; compare T_1_ lanes 4 to 6 and 7 to 9). Moreover, we saw evidence of structure changes around the RydC binding site when the oligonucleotide was annealed to the Hfq binding site on *cfa* mRNA. RNase E cleaves between residue G at position –100 and residue A at position –99. The band representing the G residue at position −101 disappeared in the oligonucleotide-bound form (Fig. S6B, compare T_1_ lanes 4 to 6 and 7 to 9). The accessibility of other residues in the vicinity of the RNase E cleavage site is also changed when oligo H1 binds to *cfa* mRNA [Fig. S6B, compare Pb(II) lanes 10 to 12 with lanes 13 to 15]. Oligo H1 binding to *cfa* mRNA could not substitute for Hfq with respect to promoting sRNA binding. Nevertheless, this *in vitro* footprinting experiment (Fig. S6B) and *in vivo* genetic analysis of interactions between the Hfq binding site (H4 mutation) (Fig. 6C and D) and the RNase E cleavage site (G3 mutation) (Fig. 5C and 6D) both support the model that structural rearrangements caused by binding of Hfq (and CpxQ) can promote changes in accessibility around the RNase E cleavage site and thus modulate *cfa* mRNA stability.

### Posttranscriptional regulation of *cfa* mRNA by sRNAs changes membrane lipid composition.

**FIG 7:**
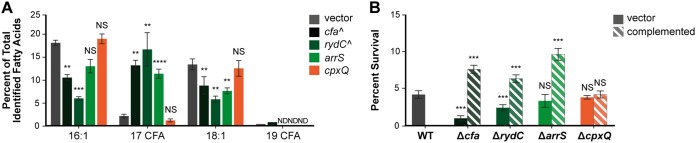
Expression of sRNAs can alter fatty acid composition and survival of acid shock. (A) Relative qualification of fatty acids in E. coli in response to ectopic expression of the vector control, *cfa*, *rydC*, *arrS*, or *cpxQ*. Fatty acids are presented as a percentage of the total identified fatty acids. Bars represent averages ± standard deviations (*n* = 3 or *n* = 2 [^]). ND, not detected. Statistical significance was determined using Student's *t* test. **, *P* < 0.005; ****, *P* < 0.0001; NS, not significant. Student's *t* test was performed by comparing the amount of each fatty acid in response to the expression of the sRNA to the fatty acid amount in the vector control. (B) Survival during an acid challenge. When strains reached an OD_600_ of 0.2, cultures were diluted into LB (pH 3) and incubated for 60 min. Survival was determined based on the ratio of the number of CFU on the LB plates after acid shock to the number of CFU on the LB plate before acid shock. Bars represent averages ± standard deviations of the results of three technical replicates. Statistical significance was determined using Student's *t* test. ***, *P < *0.0005; NS, not significant. Student's *t* test was performed by comparing the survival of each mutant to acid challenge to the survival of the vector control.

We next wanted to assess the consequences of multiple sRNAs regulating *cfa* expression with regard to the physiology of the bacterium. CFA synthase forms CFAs by transferring a methylene group from *S*-adenosylmethionine to the double bond of a UFA of a mature phospholipid that is already incorporated in the membrane. Specifically, palmitoleic acid (C_16:1_) is converted into methylene-hexadecanoic acid (C_17_CFA) and vaccenic acid (C_18:1_) is converted into methylene-octadecanoic acid (C_19_CFA). To test how posttranscriptional regulation of *cfa* by sRNAs alters the proportion of CFAs in membrane fatty acids, we conducted gas chromatography on membrane lipids isolated from strains in which each sRNA was ectopically expressed (Fig. 7A; Table S2). The fatty acid (FA) compositions of all strains were similar except for the CFA and UFA content (Fig. 7A; Table S2). In strains carrying P*_lac_-cfa*, we detected reduced levels of 16:1 and 18:1 UFAs and a ∼6-fold increase in levels of C_17_CFA, compared to levels in the strain carrying the vector control. Similarly, ArrS-producing cells had ∼5-fold higher levels of C_17_CFA and lower levels of 16:1 and 18:1 UFAs than the vector control. RydC-producing cells likewise had reduced levels of 16:1 and 18:1 UFAs and ∼8-fold increased levels of C_17_CFA compared to the control strain. CpxQ-producing strains had modestly reduced C_17_CFA (∼2-fold lower than the vector control). These data show that regulation of *cfa* mRNA translation by each sRNA is correlated with changes in membrane CFA content, implying that the regulation by sRNAs could contribute to meaningful changes in cell membrane structure and function.

### Role of sRNAs in surviving acid shock.

Because CFAs have been implicated in resistance to acidic pH (5, 6), we next investigated whether sRNAs promote survival after acid shock. Survival of wild-type, Δ*cfa*, Δ*rydC*, Δ*arrS*, and Δ*cpxQ* strains and complemented mutants was measured after a rapid shift of cultures from pH 7 to pH 3. Survival was determined based on the ratio of the number of CFU recovered after acid shock to the number of CFU before acid shock (Fig. 7B). The Δ*rydC* and Δ*cfa* strains had lower survival rates than the wild type after the acid shock. The Δ*cfa* mutant had the lowest survival rate of all the strains tested (Δ*cfa* mutant, 1.0% ± 0.3% survival versus 4.3% ± 0.5% for the wild type). The Δ*rydC* strain had a survival rate intermediate between that of the Δ*cfa* mutant and the wild type (Δ*rydC* mutant, 2.5% ± 0.4%), suggesting that RydC might be important for activating *cfa* or other targets to promote resistance to acid shock. The Δ*arrS* and Δ*cpxQ* strains showed the same survival rates as the wild type, implying that these two sRNAs do not play an important role in regulating *cfa* or other targets to promote acid shock resistance under these conditions.

Complementation of the *cfa, rydC*, and *arrS* mutations by expression of the corresponding gene from a plasmid resulted in enhanced acid shock survival compared to that of the wild-type strain (Fig. 7B, hatched bars). Enhanced acid resistance for ArrS-producing strains was observed previously and was attributed to ArrS positively regulating *gadE* expression (18). Our data suggest that ArrS-dependent activation of *cfa* might also contribute to enhanced acid resistance of ArrS-producing cells. The complemented *cpxQ* strain showed survival rates comparable to that of the wild-type strain (Fig. 7B). We have shown that ArrS and RydC both activate *cfa* posttranscriptionally and increase the CFA content in cell membranes, but only deletion of *rydC* renders otherwise wild-type cells more susceptible to acid shock. These results suggest that at least RydC-mediated activation of *cfa* translation promotes cell survival during an acid shock.

### sRNA-dependent regulation of *cfa* at acidic pH.

**FIG 8:**
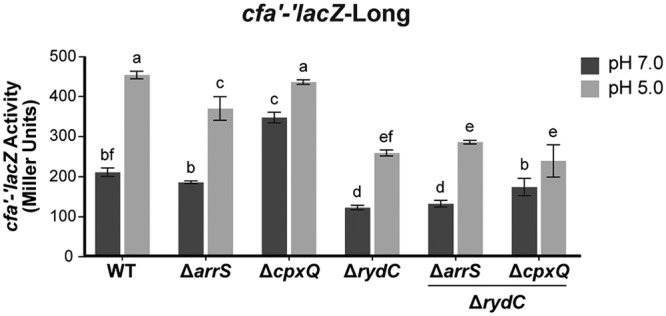
*cfa* translation is induced at acidic pH. Cells carrying *cfa'-'lacZ*-Long in either the WT background, a background where one sRNA is deleted, or a background where *rydC* and one other sRNA are deleted were grown in medium at pH 7 and then subcultured into medium at either pH 7 (pH 7.0) or pH 5 (pH 5.0). Samples were harvested 120 min later and assayed for β-galactosidase activity of the reporter fusion. Error bars represent standard deviations of the results of three biological replicates. A one‐way analysis of variance (ANOVA) with *post hoc* Tukey's test was performed; bars with the same letter at the top indicate no significant differences between the fusion activities (*P ≤ *0.05).

To further investigate sRNA-dependent regulation during acid stress, we examined sRNA-dependent regulation of *cfa* translational fusions at neutral and acidic pH values. For these experiments, we used a mild form of acid stress (pH 5) that does not kill cells so we could monitor effects on gene expression. Cells carrying *cfa'-'lacZ*-Long (Fig. 8), *cfa'-'lacZ*-Short, or *cfa'-'lacZ*-ΔsRNABS (Fig. S7A and B) were grown at either pH 7 or pH 5 and assayed for β-galactosidase activity. For *cfa'-'lacZ*-Long, activity was higher at pH 5 than at pH 7 (Fig. 8). The activities of *cfa'-'lacZ*-Short and *cfa'-'lacZ*-ΔsRNABS were similar at both pH 5 and pH 7 (Fig. S7A and B). These observations indicate that activation of *cfa* mRNA in response to acidic pH occurs posttranscriptionally, and the loss of regulation of a fusion lacking the sRNA binding sites suggests that one or more sRNAs could be responsible for regulation under these conditions.

To further probe the effects of sRNA regulation of *cfa* mRNA at acidic pH, the experiment was performed in wild-type and sRNA mutant backgrounds (Fig. 8). The activity of *cfa'-'lacZ*-Short was not affected by changes in pH or by deletion of any of the sRNAs (Fig. S7C and D). For *cfa'-'lacZ*-Long, the Δ*arrS* strain had activities at pH 7 and pH 5 similar to those of the wild type, with activity at pH 5 higher than that at pH 7 (Fig. 8). The Δ*cpxQ* mutant had higher *cfa'-'lacZ* activity at pH 7 than the wild type (Fig. 8). Since ectopic expression of *cpxQ* repressed *cfa* translation (Fig. 1B), the increased levels of *cfa'-'lacZ* activity in the Δ*cpxQ* mutant suggests that at neutral pH, CpxQ is produced at sufficient levels to repress *cfa*. In contrast, at pH 5, the Δ*cpxQ* mutant and the wild type had similar levels of *cfa'-'lacZ*-Long activity (Fig. 8). A Northern blot analysis to measure CpxQ levels at pH 5 and pH 7 determined that CpxQ levels were indeed higher at pH 7 than at pH 5 (Fig. S8). The Δ*rydC* mutant had lower *cfa'-'lacZ* activity than the wild type at both pH 7 and pH 5, suggesting that RydC may be produced at sufficient levels at both neutral and acidic pH to have an activating effect on *cfa* under both conditions. We have shown that ectopic production of ArrS or RydC activates *cfa* and increases the CFA content in cell membranes, but only deletion of *rydC* affects *cfa'-'lacZ*-Long activity at pH 5. This observation fits with our previous result that deletion of *rydC* renders otherwise wild-type (*cfa*^+^) cells more susceptible to acid shock (Fig. 7B).

To further investigate the interplay of the sRNAs on *cfa* mRNA translation at different pH values, we deleted *rydC* in combination with each of the other sRNAs in the *cfa'-'lacZ*-Long strain (Fig. 8) and measured β-galactosidase activity of the reporter fusions at pH 5 and pH 7. Deletion of *arrS* in the Δ*rydC* background had no effect on *cfa'-'lacZ*-Long activity in comparison to the Δ*rydC* parent (Fig. 8), suggesting that ArrS does not play a role in regulation of *cfa* translation under these conditions. The Δ*cpxQ* Δ*rydC* strain had slightly higher *cfa'-'lacZ*-Long activity than the Δ*rydC* parent at pH 7 (Fig. 8). In contrast, at pH 5, the Δ*rydC* parent and Δ*cpxQ* Δ*rydC* strain had similar levels of *cfa'-'lacZ*-Long activity (Fig. 8). All together, the data are consistent with the hypothesis that RydC exerts a positive effect on *cfa* mRNA at both neutral and acidic pH values under our growth conditions. In contrast, CpxQ has a mild repressive effect on *cfa* mRNA only at neutral pH.

### RpoS is not responsible for observed differences in *cfa* mRNA regulation.

**FIG 9:**
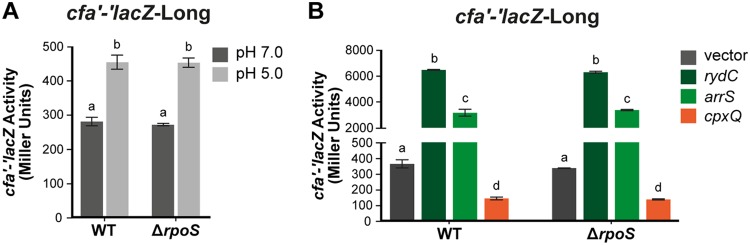
Deletion of *rpoS* does not affect *cfa'-'lacZ*-Long activity. (A) Cells carrying *cfa'-'lacZ*-Long in a WT or Δ*rpoS* background were grown, and *cfa* translation in response to acid was assayed as described in the legend for Fig. 8. The error bars represent standard deviations of the results of three independent experiments, and statistical significance was determined using two-tailed Student's *t* test. (B) *cfa'-'lacZ*-Long in a WT or Δ*rpoS* background carrying an empty, P_lac_-*arrS*, P_lac_-*cpxQ*, or P_lac_-*rydC* plasmid was treated as described in the legend for Fig. 1B. Statistical significance was determined using two-tailed Student's *t* test. See the legend for Fig. 8 for an explanation of the letters at the top of the bars.

A previous study suggested that transcription from the σ^s^-dependent *cfa* promoter was increased when cells were grown at pH 5 compared to when grown at pH 7 (6). Our *cfa'-'lacZ*-Long reporter fusion contains the region encompassing the σ^s^-dependent promoter. To determine if the increased *cfa'-'lacZ*-Long activity we observed during mild acid stress was dependent on RpoS, we measured *cfa'-'lacZ*–Long activity at pH 5 and pH 7 in a Δ*rpoS* background (Fig. 9A). Deletion of *rpoS* had no effect on *cfa'-'lacZ*-Long activity at pH 5 or pH 7, and we observed the same increase in activity in response to pH 5 in both wild-type and Δ*rpoS* backgrounds (Fig. 9A), indicating that RpoS does not impact the observed activity of the *cfa'-'lacZ*-Long fusion.

We showed that ectopic production of each sRNA, RydC, ArrS, and CpxQ, can regulate *cfa'-'lacZ*-Long (Fig. 1B). While the experiment described above suggests that pH-dependent regulatory effects are not mediated by RpoS, we wanted to further test whether sRNAs could be acting indirectly on *cfa* via posttranscriptional regulation of *rpoS*. We ectopically expressed RydC, ArrS, or CpxQ in wild-type and Δ*rpoS cfa'-'lacZ*-Long strains and did not observe any differences in sRNA-dependent regulation between wild-type and Δ*rpoS* strains (Fig. 9B), indicating that the sRNA-dependent regulation is not mediated indirectly through RpoS. These data further support the idea that the regulation of *cfa* by these sRNAs is direct.

## DISCUSSION

**FIG 10:**
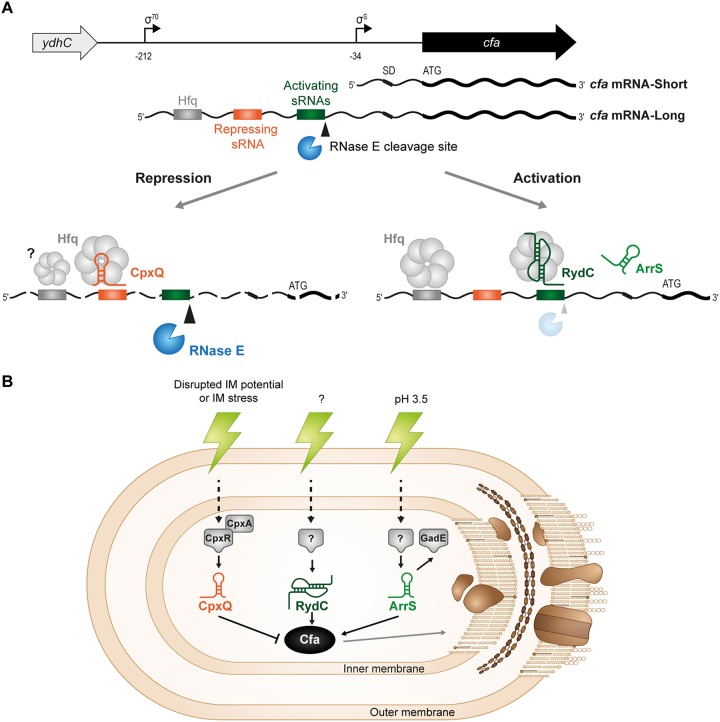
Models of sRNA regulation of *cfa* mRNA. (A) Working model for the role of Hfq, RNase E, and sRNAs in the regulation of *cfa* mRNA. There are multiple RNase E cleavage sites that have been mapped in *Salmonella*. Activating sRNAs, especially RydC, require Hfq for efficient pairing and inhibition of RNase E cleavage at one site that may be the primary cleavage site, and the pairing stabilizes *cfa* mRNA. CpxQ may have the opposite effect: somehow pairing of CpxQ (facilitated by Hfq) stimulates RNase E-dependent cleavage at the site RydC is known to protect. (B) Regulation of *cfa* mRNA by these sRNAs provides a σ^S^-independent mechanism for control of *cfa* and thus a way to modulate the lipid composition of the bacterial cell envelope in response to a variety of different stresses. CpxQ is part of the Cpx regulon, which responds to signals like disrupted inner membrane potential or inner membrane stress. The conditions that stimulate RydC production are unknown. ArrS has been linked to acid stress, and CFAs have previously been implicated in acid resistance. Modulating the concentration of cyclopropane fatty acids in response to these stresses might alter membrane properties like fluidity and permeability and allow the cell to adapt to the stress.

Microbial membranes are the first line of defense against environmental stress, as well as the site of many metabolic processes. As such, maintenance of membrane integrity and homeostasis is key to cell survival. sRNAs are important posttranscriptional regulators that play key roles in the response to environmental stress. We are interested in how sRNAs can contribute to membrane modification in response to stressors, particularly how sRNAs can lead to altered membrane phospholipid composition. In the present work, we investigated the roles of multiple sRNAs in posttranscriptional regulation of *cfa* mRNA, encoding a key enzyme used to alter lipid composition of bacterial membranes. We show that *cfa* can be posttranscriptionally regulated by three sRNAs, all of which act on the same long isoform of *cfa* mRNA that results from transcription at the constitutive σ^70^-dependent promoter (Fig. 10A). RydC and ArrS posttranscriptionally activate *cfa*, while the sRNA CpxQ represses *cfa* (Fig. 1B). RydC and ArrS bind at a site that overlaps an RNase E cleavage site to prevent RNase E cleavage, increase *cfa* mRNA stability, and allow for increased *cfa* translation. CpxQ represses *cfa* mRNA by modulating cleavage at the same RNase E cleavage site that is protected by RydC and ArrS (Fig. 5). An Hfq binding site near the 5′ end of the *cfa* mRNA long isoform affects *cfa* mRNA stability (Fig. 6D) and is required for CpxQ-mediated repression (Fig. 6C and D). Our results are consistent with the model that Hfq promotes CpxQ base pairing with *cfa* mRNA and makes the downstream RNase E cleavage site more accessible, thereby promoting *cfa* mRNA degradation (Fig. 10A).

Hfq is clearly a key player in sRNA-dependent activation and repression of *cfa*. RydC requires Hfq to stabilize *cfa* mRNA (11), but this activity does not require the identified 5′-end Hfq binding site (H4 mutation) (Fig. 6C and D), indicating that there may be another Hfq binding site in the 5′ UTR of *cfa*. Activating and repressing sRNAs may direct alternative sites or modes of Hfq binding and in turn modulate mRNA structure and accessibility of the RNase E cleavage site. *In vitro* footprinting did not reveal an Hfq-dependent protection of *cfa* mRNA (Fig. 2B). Stable binding and clear protection may not be seen if alternative binding sites are competing for Hfq *in vitro*. DNA oligo H1-mediated protection of the 5′-localized Hfq binding site could not promote sRNA binding to *cfa* mRNA in the absence of Hfq (Fig. S6B). Hfq CLIP-seq in *Salmonella* (26) revealed a potential second Hfq-*cfa* mRNA interaction site located directly upstream of the RydC base-pairing site and overlapping the CpxQ base-pairing site. However, the cross-linking-induced mutation was not determined, so the key nucleotide determinants of this additional putative Hfq binding site are not yet known.

Regulation of *cfa* by multiple sRNAs suggests that *cfa* mRNA is another example of an mRNA that acts as a regulatory hub for environmental signal integration via sRNAs. Other examples include *rpoS* mRNA, which is activated by multiple sRNAs (28–30), and *flhDC* mRNA, which is repressed by multiple sRNAs (31). We hypothesize that the sRNAs regulating *cfa* mRNA are each produced under a distinct stress condition and that their regulation of *cfa* mRNA under each condition enhances stress adaptation or survival (Fig. 10B). We found that while ectopic production of the activating sRNAs (RydC and ArrS) could promote enhanced survival under acid stress conditions, only the *rydC* mutant had an acid sensitivity phenotype (Fig. 7B). This is consistent with the idea that while RydC-mediated regulation of *cfa* enhances the response to acid stress, ArrS and CpxQ may be important for regulating CFA levels under other stress conditions. Uncovering the signals and regulators controlling synthesis of these sRNAs, particularly RydC and ArrS, will provide new clues regarding other stress conditions where regulation of membrane CFAs may be physiologically relevant (Fig. 10B).

CpxQ is the noncoding regulator of the inner membrane stress response mediated by the CpxAR two-component system. In *Salmonella*, CpxQ can mitigate growth inhibition caused by exposure to the protonophore CCCP (carbonyl cyanide *m*-chlorophenylhydrazone), which dissipates inner membrane potential (15, 16). CpxQ repression of *nhaB* mRNA, which encodes an inner membrane sodium-proton antiporter, is particularly important for this phenotype. It is possible that CpxQ regulation of *cfa* also helps to maintain inner membrane potential, but the effect of CFAs on membrane potential is currently unknown. A previous study showed that a lack of CFAs in cellular membranes increases H^+^ permeability but decreases H^+^ extrusion (4). Furthermore, the Cpx response is widely thought to be important for the regulation of energy production and substrate transport across the inner membrane (32) and fatty acid composition is known to affect inner membrane transporter activity and protein function (33, 34), but how CFAs affect these functions has not been studied. However, there is a linear correlation between membrane protein activity and membrane fluidity (34), and, *in vitro*, CFAs are known to increase membrane fluidity by disrupting lipid packing (35). Another possibility that has not been explored is the effect of the Cfa protein and mechanism of catalysis on inner membrane integrity. During catalysis, one subunit of the Cfa dimer binds to the membrane while the other subunit extracts and modifies unsaturated lipids from the membrane (36). Given that the Cpx response regulates events at the inner membrane and predominantly inhibits the production of inner membrane-localized protein complexes, the tight association and interaction of Cfa with the inner leaflet of the cytoplasmic membrane may trigger the Cpx response. Perhaps CpxQ acts by a feedback inhibition mechanism to reduce the membrane stress associated with Cfa enzymatic activity. Finally, proton motive force (PMF) is the cellular energy source, and the conversion of UFAs to CFAs by Cfa is an energetically expensive reaction (8). Thus, CpxQ may repress *cfa* translation when PMF is dissipated simply to reduce cellular ATP consumption. Clearly defining the impact of Cfa on PMF and determining under what conditions CpxQ differentially regulates its regulon would elucidate the physiological function of CpxQ regulation of *cfa*.

Extensive previous work has shown that sRNAs play an integral role in changing membrane protein composition in response to changing environments (37–40), but how sRNAs influence membrane fatty acid composition is not well studied. Studies of RydC structure (11–13), interaction with Hfq (41), and regulation of mRNA targets (11–14) have hinted that this sRNA might be important for regulating cell envelope structure and function, yet the physiological role of RydC has remained enigmatic. Our detailed characterization of sRNA-mediated control of *cfa* expression led us to discover the first phenotype for *rydC* mutants—sensitivity to acid shock (Fig. 7B). The link between membrane CFAs and acid resistance was made decades ago but was attributed solely to RpoS-mediated transcriptional regulation (6). Our study reveals that a previously undiscovered level of posttranscriptional regulation of *cfa* also contributes to the acid stress response. Our work demonstrates that sRNAs contribute to the modulation of the cell envelopes of bacteria not only by changing membrane protein composition but also by directly modulating membrane lipid composition, with implications for membrane stability and fluidity.

## MATERIALS AND METHODS

### Strain and plasmid construction.

Strains and plasmids used in this study are listed in Table S3 in the supplemental material. All strains used in this study are derivatives of E. coli K-12 DJ624 (D. Jin, National Cancer Institute). Oligonucleotide primers used in this study are listed in Table S4. Integrated DNA Technologies or Sigma-Aldrich synthesized all primers. Δ*rpoS* and Δ*cfa* mutations were made via P1 *vir* transduction from the Keio collection (42). All other chromosomal mutations were made using λ Red recombination (43, 44), and marked alleles were moved between strains by P1 *vir* transduction (45). Kanamycin markers were removed using pCP20 (43).

The *lacZ* translational reporter fusions were constructed using λ Red homologous recombination into strain PM1205 and counterselection against *sacB*, as previously described (46). Transcription of the fusion is under the control of the P_BAD_ promoter. The fusion contains the *cfa* 5′ UTR fragment of interest and the first 36 codons of the *cfa* open reading frame. *cfa'-'lacZ*-Long, *cfa'-'lacZ*-Short, *cfa'-'lacZ*-ΔsRNABS, and *cfa'-'lacZ*-ΔHfqBS were constructed by PCR, amplifying the *cfa* 5′ UTR fragment of interest from DJ624 using a fusion-specific forward primer containing flanking homology to P_BAD_ and the reverse primer O-AK24R, which contains flanking homology to *lacZ* and anneals in the *cfa* coding region (Table S4). *cfa'-'lacZ*-Long and *cfa'-'lacZ*-Short were constructed by A. M. King et al. (14). *cfa'-'lacZ*-LongH1 and -H4 were constructed by using a forward primer containing the desired point mutations and flanking homology to P_BAD_ and reverse primer O-AK24R to PCR amplify the *cfa* 5′ UTR fragment of interest from DJ624 (Table S4). *cfa'-'lacZ*-LongM1 was made using Gibson Assembly (47). The genes *amp* and *ori* of pBR322 were PCR amplified using primers with 5′ homologies to the *cfa* 5′ UTR containing the desired mutations. The Gibson reaction was performed using NEBuilder HiFi DNA assembly master mix (New England Biolabs) according to the manufacturer’s protocol. The Gibson product was transformed into XL10 competent cells, and the plasmid was purified. The mutated *cfa* 5′ UTR was PCR amplified from this plasmid using the same primers used to create *cfa'-'lacZ*-Long. The plasmid used to make *cfa'-'lacZ*-LongM1, which contains the G101C point mutation, was saved as pCB1 for further strain construction. The *cfa'-'lacZ*-LongG1 and -G2 RNase E recognition site mutant fusions were made by mutating pCB1 using QuikChange mutagenesis (Agilent Technologies) and oligonucleotides that restore the G101C mutation to the WT and add the additional desired mutations. The plasmid used to make *cfa'-'lacZ*-LongG2 was saved as pCB2. *cfa'-'lacZ*-LongG4 was made by mutating pCB2 using QuikChange mutagenesis (Agilent Technologies) and oligonucleotides that add the desired mutations. The mutated *cfa* 5′ UTR was PCR amplified from each plasmid using the same primers used to create *cfa'-'lacZ*-Long. To make *cfa'-'lacZ*-LongM2 and *cfa'-'lacZ*-LongH4G3, gBlocks gene fragments containing the desired mutations were ordered from Integrated DNA Technologies. All PCR products or gBlocks gene fragments were recombined into PM1205 using λ Red homologous recombination and counterselection against *sacB* as previously described (46). All fusions were verified using DNA sequencing.

Plasmids containing WT sRNAs, mutant sRNAs, and *cfa* under the control of the P_LlacO_ promoter were constructed by PCR amplifying each sRNA from E. coli DJ624 chromosomal DNA using oligonucleotides containing HindIII and BamHI restriction sites (Table S4). The primers used to create P_LlacO_-*arrSM1*, P_LlacO_-*rydCM1*, and P_LlacO_-*cpxQM2* contained the desired point mutations. PCR products and vector pBRCS12 (48) were digested with HindIII and BamHI (New England Biolabs) restriction endonucleases. Digestion products were ligated using DNA ligase (New England Biolabs), the plasmids were transformed into XL10 competent cells, and the plasmid was purified. All plasmids were confirmed by DNA sequencing.

### Media and growth conditions.

Bacteria were cultured in LB broth medium or on LB agar plates at 37°C, unless stated otherwise. When necessary, media were supplemented with antibiotics at the following concentrations: 100 μg ml^−1^ ampicillin (Amp) or 25 μg ml^−1^ kanamycin (Kan). Isopropyl-β-d-1-thiogalactopyranoside (IPTG) was used at 0.1 mM (final concentration) for induction of expression from the P_LlacO-1_ promoter.

### β-Galactosidase assays. (i) Ectopic expression of sRNAs.

Bacterial strains harboring translational *lacZ* reporter fusions carrying the sRNA plasmid were cultured overnight in Terrific broth (TB) with Amp and 0.002% l-arabinose and then subcultured 1:100 into fresh TB medium containing Amp and 0.002% l-arabinose. Strains were grown at 37°C with shaking to early exponential phase, and then 0.1 mM IPTG was added to induce sRNA expression. Samples were harvested 60 min later, and β-galactosidase assays were then performed as previously described (45).

### (ii) Acid stress.

Bacterial strains harboring translational *lacZ* reporter fusions were cultured overnight in TB medium (pH 7.0) with 0.002% l-arabinose and then subcultured 1:100 into fresh TB medium (pH 7.0) containing 0.002% l-arabinose. Strains were grown at 37°C with shaking for 2 h and then subcultured 1:100 again into TB medium with 0.002% l-arabinose at pH 7.0 or pH 5.0. Samples were harvested 120 min later, and β-galactosidase assays were then performed as previously described (45).

### Northern blot analysis.

To measure CpxQ expression from CpxQ, CpxQ1, and CpxQ2 plasmids, bacterial strains were cultured in LB medium to an optical density at 600 nm (OD_600_) of ∼0.4, and then plasmids were induced with 0.1 mM IPTG for 60 min. To measure CpxQ expression in response to pH 5 and pH 7, the wild-type strain, DJ624, was grown in TB medium at pH 7 and then subcultured into TB medium at either pH 7 or pH 5 and grown for 2 h. For both experiments, RNA was extracted using the hot phenol method as described previously (49). RNA concentrations were measured spectrophotometrically. Fifteen micrograms of RNA was denatured for 5 min at 95°C in loading buffer (containing 95% formamide), separated on an 8% polyacrylamide urea gel at 100 V for 1 h using 1× Tris-acetate-EDTA (TAE), and then transferred to a BrightStar-Plus positively charged nylon membrane in 0.5× TAE buffer by electroblotting at 50 V for 1 h at 4°C. RNA was cross-linked to the membrane, and then the membrane was prehybridized for 45 min in ULTRAhyb (Ambion) solution at 42°C. Blots were hybridized overnight with ^32^P-end-labeled primer CpxQNB, and then membranes were washed at 42°C twice with 2× SSC–0.1% SDS for 8 min and then twice with 0.1× SSC–0.1% SDS for 15 min. The signal was detected using film. Membranes were stripped in boiling 0.1% SDS for 10 min and then reprobed overnight with ^32^P-end-labeled primer 5S. The ladder was prepared using the Decade markers system from ThermoFisher, according to the manufacturer’s instructions.

### Analysis of fatty acids.

Cells were cultured overnight in LB containing Amp and subcultured 1:100 into fresh LB containing Amp. Cells were grown to an OD_600_ of 0.1, and then plasmids were induced with 0.1 mM IPTG for 1 h. Cells were then spun down, and the cell pellet was washed twice with MilliQ water. Fatty acids were extracted according to a previously described protocol, method 3.1 (50). Pentadecanoic acid methyl ester was used as an internal standard. The bacterial sample was spiked with 500 μg of the internal standard, and then the sample was transesterified with 2 ml of 0.5 M sodium methoxide for 1 min at room temperature. Fatty acid methyl esters (FAMEs) were then extracted using 2 ml of hexane. The solution was centrifuged at 2,000 rpm for 5 min, and the organic upper phase was removed and dried under nitrogen. FAMEs were treated with trimethylsilyldiazomethane in methanol to ensure complete methylation. FAMEs were identified on an Agilent 6890N gas chromatograph (GC) with a 5973 mass spectrometer (MS) and a Zebron WAX column (30 m by 0.25 mm by 0.25 μm).

### Acid shock assay.

Strains were grown overnight in LB with Amp, subcultured 1:100 into LB with Amp at pH 7.0, and grown at 37°C with shaking until the OD_600_ reached 0.1. Plasmids were induced with 0.1 mM IPTG. When strains reached an OD_600_ of 0.2, cultures were diluted 10-fold into LB (pH 3.0) and incubated at 37°C with no shaking for 60 min. Survival was determined based on the ratio of the number of CFU on the LB plates after acid shock to the number of CFU on the LB plate before acid shock.

### *In vitro* RNA synthesis and structure probing.

DNA templates carrying a T7 promoter sequence for *in vitro* RNA synthesis were generated by PCR from E. coli genomic DNA (*cfa* mRNA, KFO-0702/KFO-0703; RydC, KFO-0704/KFO-0705; ArrS, KFO-0708/KFO-0709; GadF, KFO-0710/KFO-0711; CpxQ, KFO-0740/KFO-0707; GcvB, KFO-0880/KFO-0881; OxyS, KFO-0884/KFO-0885). Two hundred nanograms of template DNA was transcribed using the AmpliScribe T7-Flash transcription kit (Epicentre) in accordance with the manufacturer’s recommendations; the size and integrity of the transcripts were verified on a denaturing 6% polyacrylamide gel.

RNA structure probing was carried out as described previously (11) with few modifications. In brief, for Hfq/sRNA/mRNA samples, 0.4 pmol 5′-end-labeled *cfa* mRNA was mixed with 0.4 pmol E. coli Hfq protein (provided by K. Bandyra and B. F. Luisi, University of Cambridge) or Hfq dilution buffer (1× structure buffer, 1% [vol/vol] glycerol, 0.1% [vol/vol] Triton X-100) in the presence of 1× structure buffer (0.01 M Tris [pH 7], 0.1 M KCl, 0.01 M MgCl_2_) and 1 μg yeast RNA, and samples were incubated at 37°C for 10 min. Subsequently, unlabeled sRNA (4 pmol) or water was added, and reaction mixtures were kept at 37°C for an additional 10 min. Similarly, for structure probing in the presence of an oligonucleotide blocking the Hfq binding site, 0.4 pmol *cfa* mRNA was mixed with 4 pmol unlabeled sRNA (or an equal volume of water) in the presence of 1× structure buffer (0.01 M Tris [pH 7], 0.1 M KCl, 0.01 M MgCl_2_) and 1 μg yeast RNA, and samples were incubated at 37°C for 10 min. Subsequently, 4 pmol of oligo H1 (or an equal volume of water) was added, and reaction mixtures were kept at 37°C for an additional 10 min. Samples were treated with RNase T_1_ (0.1 U; Ambion no. AM2283) for 2 min or with lead(II) acetate (final concentration, 5 mM; Sigma no. 316512) for 1.5 min.

### Primer extension.

Primer extension experiments were performed as previously described (11). A 5′-end-labeled primer (KFO-0920) specific to *gfp* was used for reverse transcription of the samples, as well as for the preparation of a template-specific ladder (prepared using the USB cycle sequencing kit; Affymetrix USB no. 78500; template amplification pZE-Cat/KFO-0920 on pKF206).

## Supplementary Material

Supplemental file 1
