# Supplementary material for: Bacterial Cyclopropane Fatty Acid Synthase mRNA Is Targeted by Activating and Repressing Small RNAs
Source: J Bacteriol. 2019 Sep 6;201(19):e00461-19. doi: 10.1128/JB.00461-19 (PMC6755755; doi:10.1128/JB.00461-19)
Supplement: Supplemental file 1 [file JB.00461-19-s0001.pdf]

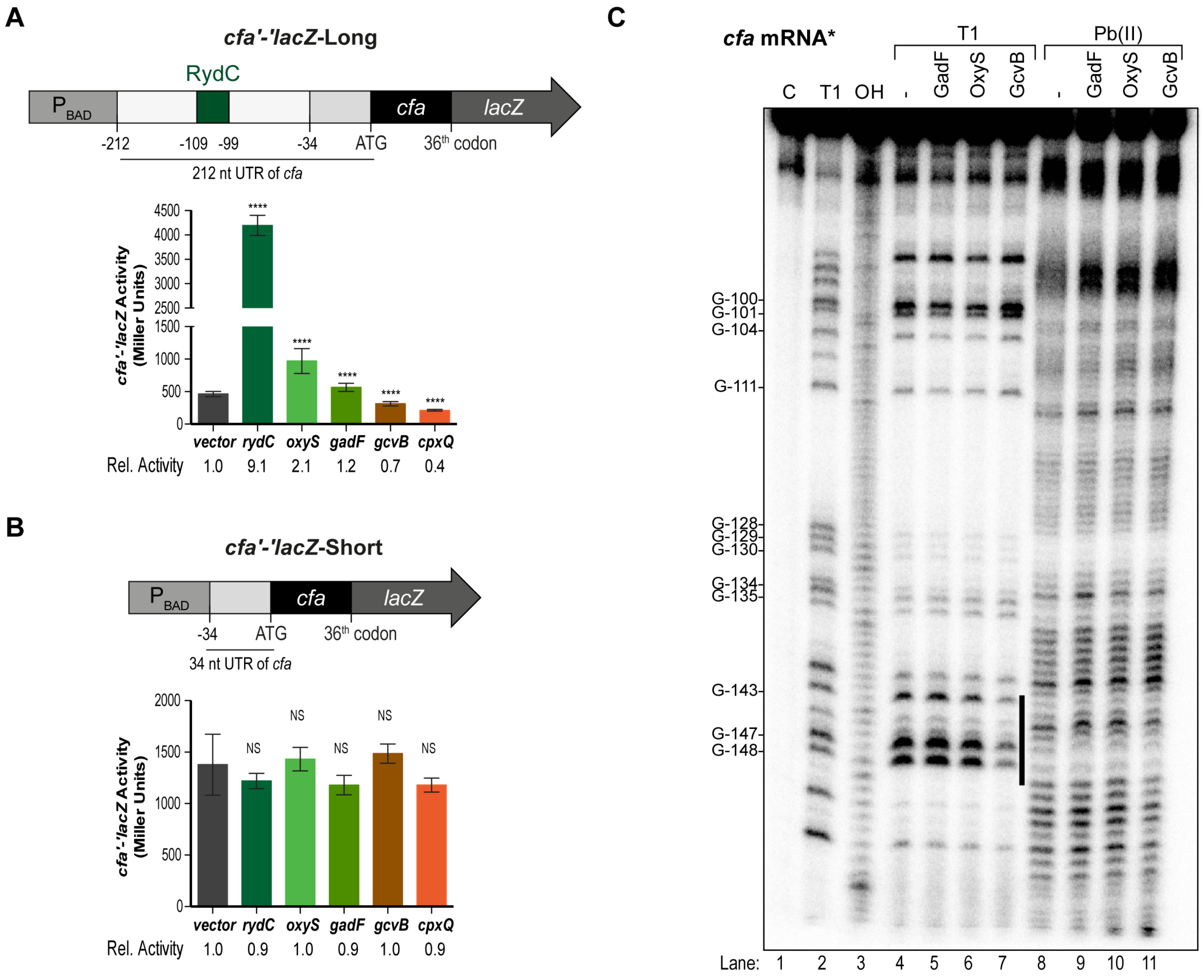

Figure S1: Testing of additional sRNAs for regulation of *cfa* expression

(A) The *cfa'-'lacZ*-Long fusion contains the entire 212-nt 5′ UTR, including the RydC binding site (indicated by green square labeled "RydC"). Regulation of *cfa'-'lacZ*-Long by RydC, OxyS, GadF, GcvB, and CpxQ was determined as described in Fig. 1B.

(B) The *cfa'-'lacZ*-Short fusion contains only proximal the σ^s-dependent promoter. Regulation of this fusion by RydC, OxyS, GadF, GcvB, and CpxQ was determined as described in Fig. 1B.

(C) *In vitro* structure probing using 5′ end-labeled *cfa* mRNA with RNase T1 (lanes 4–7) and lead(II) acetate (lanes 8–11) in the presence of Hfq (20 nM) and each sRNA (200 nM). RNase T1 and alkaline ladders of *cfa* mRNA were used to map cleaved fragments. Positions of G-residues are indicated relative to the translational start site. The GcvB binding site is marked with a black line right of the lane and was only detected in the RNase T1 digestion. The OxyS and GadF binding sites could not be detected.

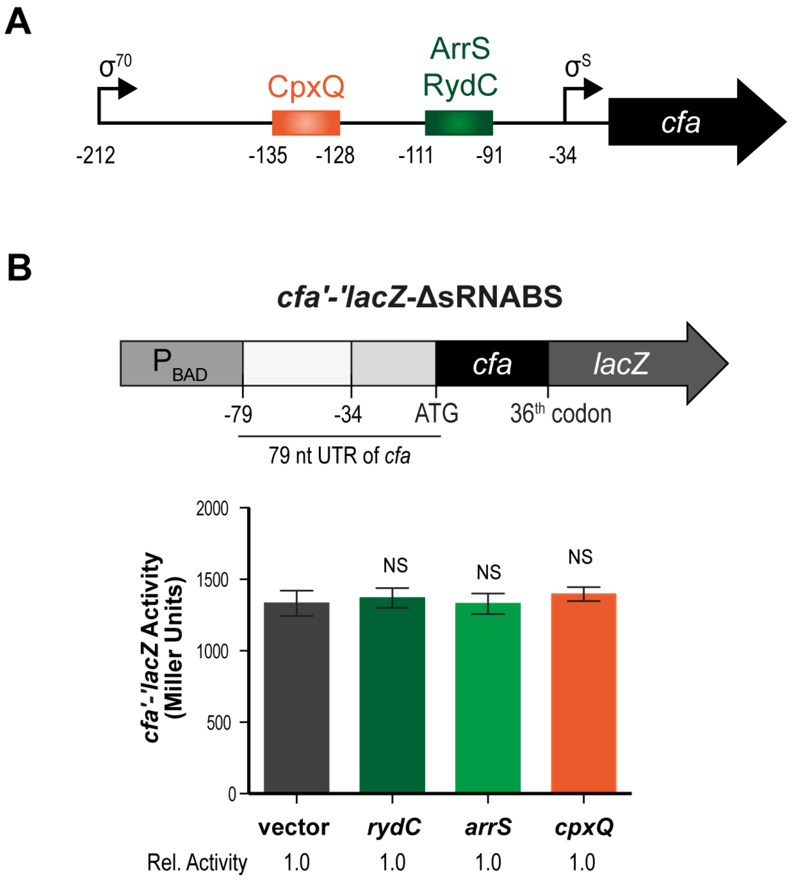

Figure S2: Deletion of the putative sRNA binding site removes regulation of *cfa* translation by ArrS, CpxQ, and RydC

(A) 5′ UTR of *cfa* gene. Arrows mark transcriptional start sites and sRNA binding sites are indicated with labeled boxes.

(B) A *cfa* translational fusion to *lacZ* (*cfa'*-*'lacZ*-ΔsRNABS) that begins immediately downstream of the predicted sRNA binding sites was constructed. This fusion contains the proximal σ^s-dependent promoter and 79-nt upstream of this promoter. Regulation of *cfa'*-*'lacZ*-ΔsRNABS by ArrS, CpxQ, and RydC was tested as described in Figure 1B.

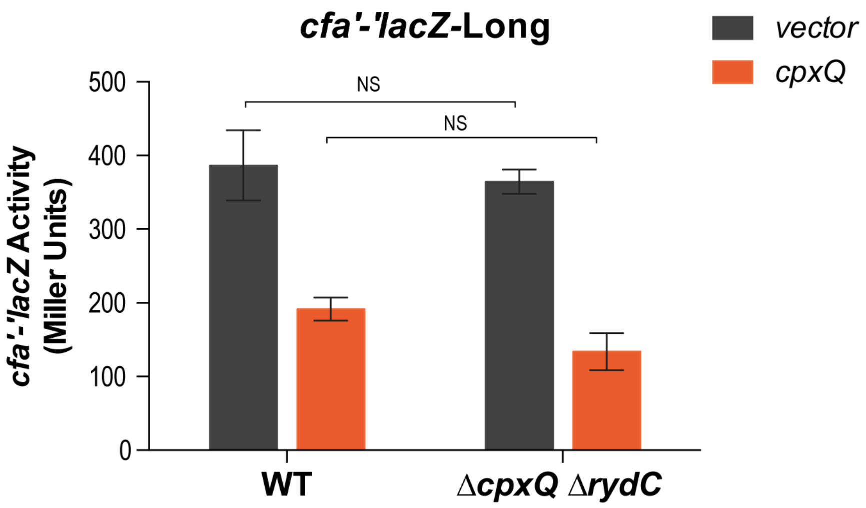

Figure S3: Deletion of *rydC* does not alter *cfa* mRNA regulation by CpxQ
CpxQ mediated repression of *cfa'-'lacZ*-Long was tested in a WT or Δ*cpxQ* Δ*rydC* background as described in Figure 1B. The statistical significance was determined using a two-tailed Student's *t*-test, ns is for not significant.

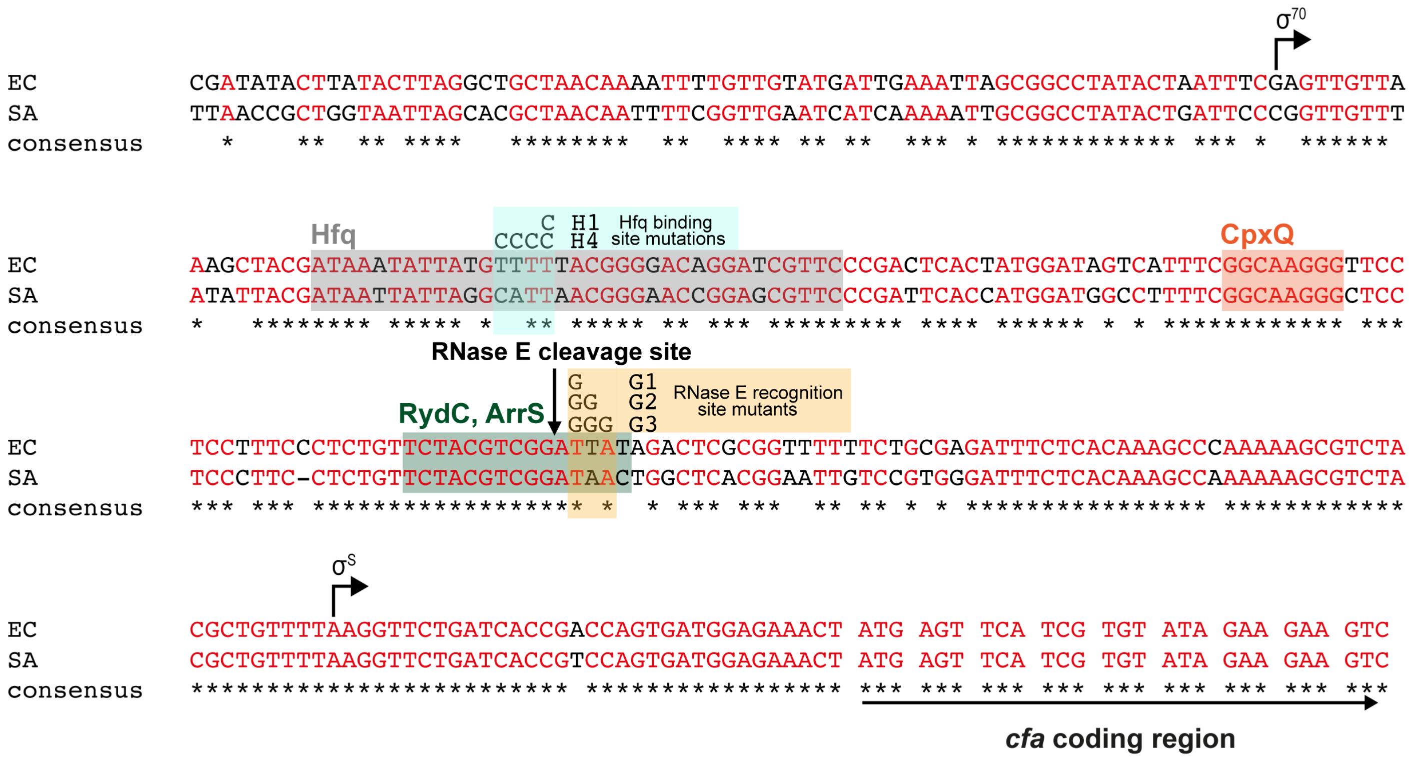

Figure S4: The *cfa* 5′ UTR differs between *E. coli* and *Salmonella*

Sequence alignment of the promoter and 5′ UTR of *cfa* in *E. coli* and *Salmonella*. Gray box: Hfq binding site. Orange box: CpxQ binding site. Green box: Activating sRNA binding site. Black arrow: RNase E cleavage site. The transcription start sites are labeled. H1 and H4 mutations in the Hfq binding site are shown in a blue shaded box. G1, G2, and G3 mutations in the RNase E recognition site are shown in a yellow shaded box.

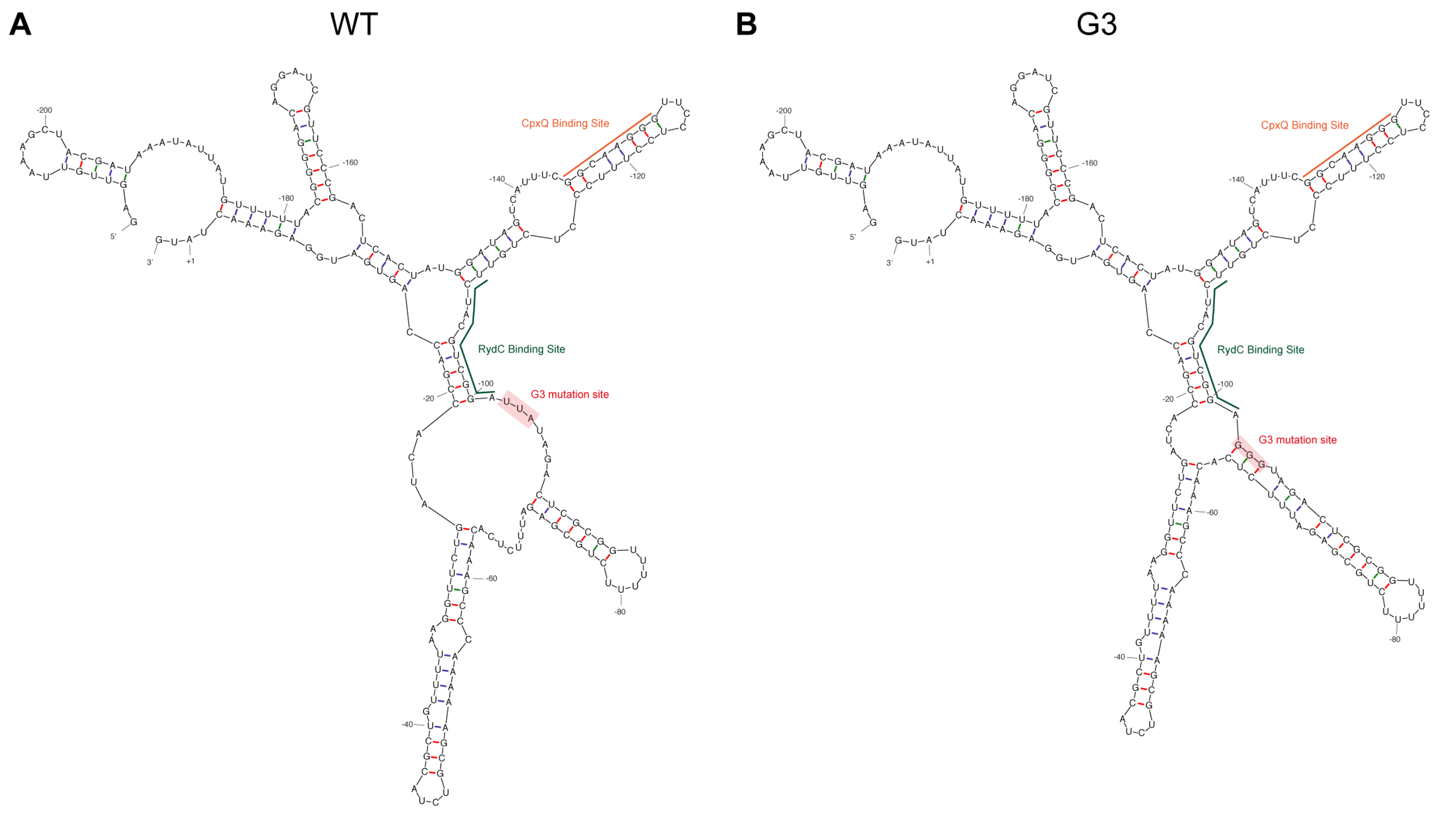

Figure S5: Structure of WT and G3 mutant *cfa* mRNA 5′ UTR

(A) Secondary stucture of the WT *cfa* mRNA 5′ UTR and (B) the G3 mutation in the *cfa* mRNA 5′ UTR as predicted by mFold. The three nucleotides mutated in the G3 mutant are in a red shaded box. The CpxQ binding site (orange) and the RydC binding site (green) are labeled. Note that the structures of the CpxQ and RydC binding sites are the same in G3 mutant as in the WT. Numbered nucleotides are relative to the translational start site (labeled +1).

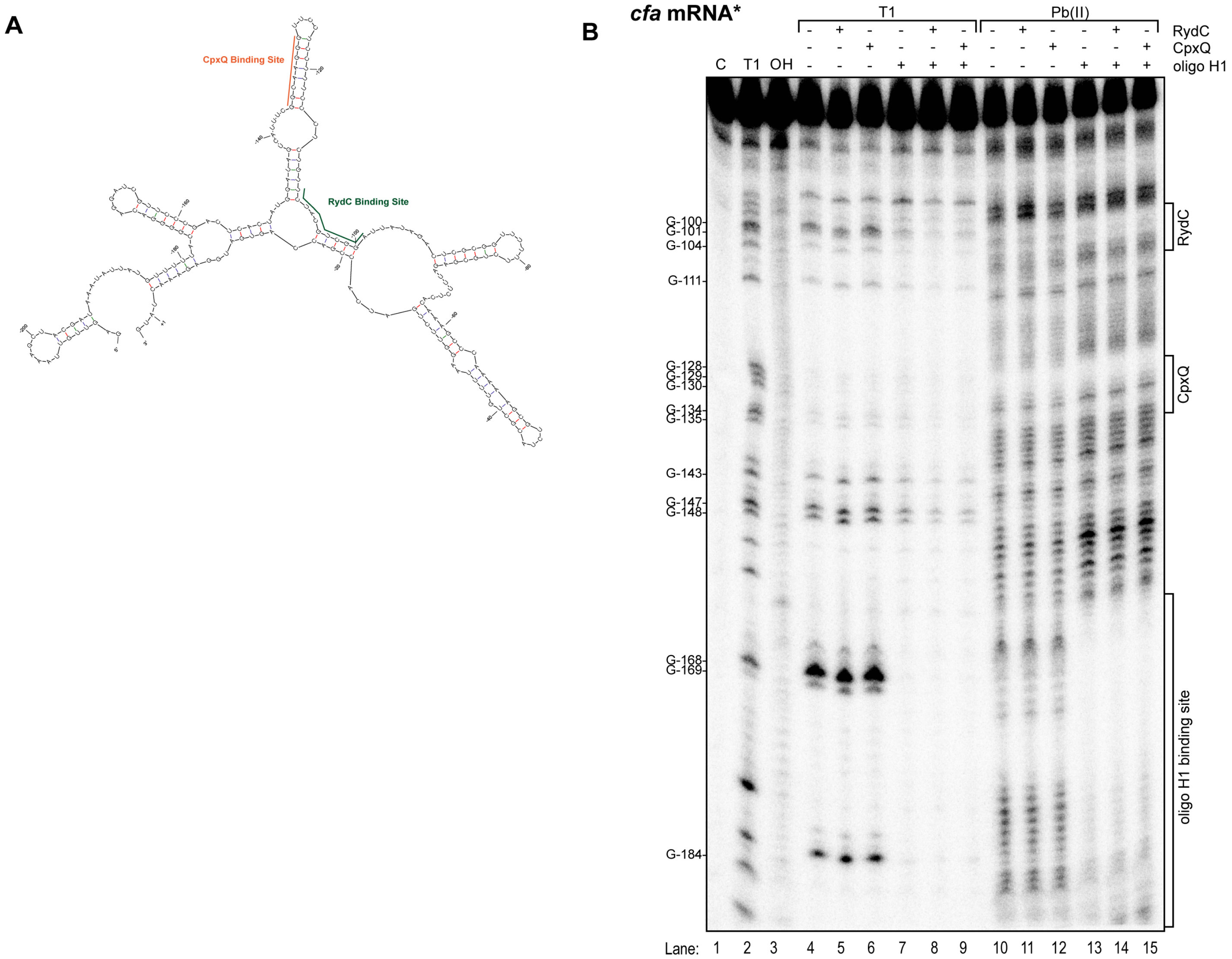

Figure S6: DNA-oligo annealing to Hfq binding region does not alter sRNA binding

(A) Secondary stucture of the *cfa* mRNA 5′ UTR as predicted by mFold. The CpxQ binding site (orange) and the RydC binding site (green) are labeled. Numbered nucleotides are relative to the translational start site (labeled +1).

(B) *In vitro* structure probing using 5′ end-labeled *cfa* mRNA with RNase T1 (lanes 4–9) and lead(II) acetate (lanes 10–15) in the presence of a ssDNA-oligo (oligo H1) that base-pairs to the Hfq binding region (-195 to -161) in the 5′ UTR of *cfa* mRNA and either CpxQ or RydC. RNase T1 and alkaline ladders of *cfa* mRNA were used to map cleaved fragments. Positions of G-residues are indicated relative to the translational start site. The RydC, CpxQ, and oligo H1 base-pairing sites are labeled on the right.

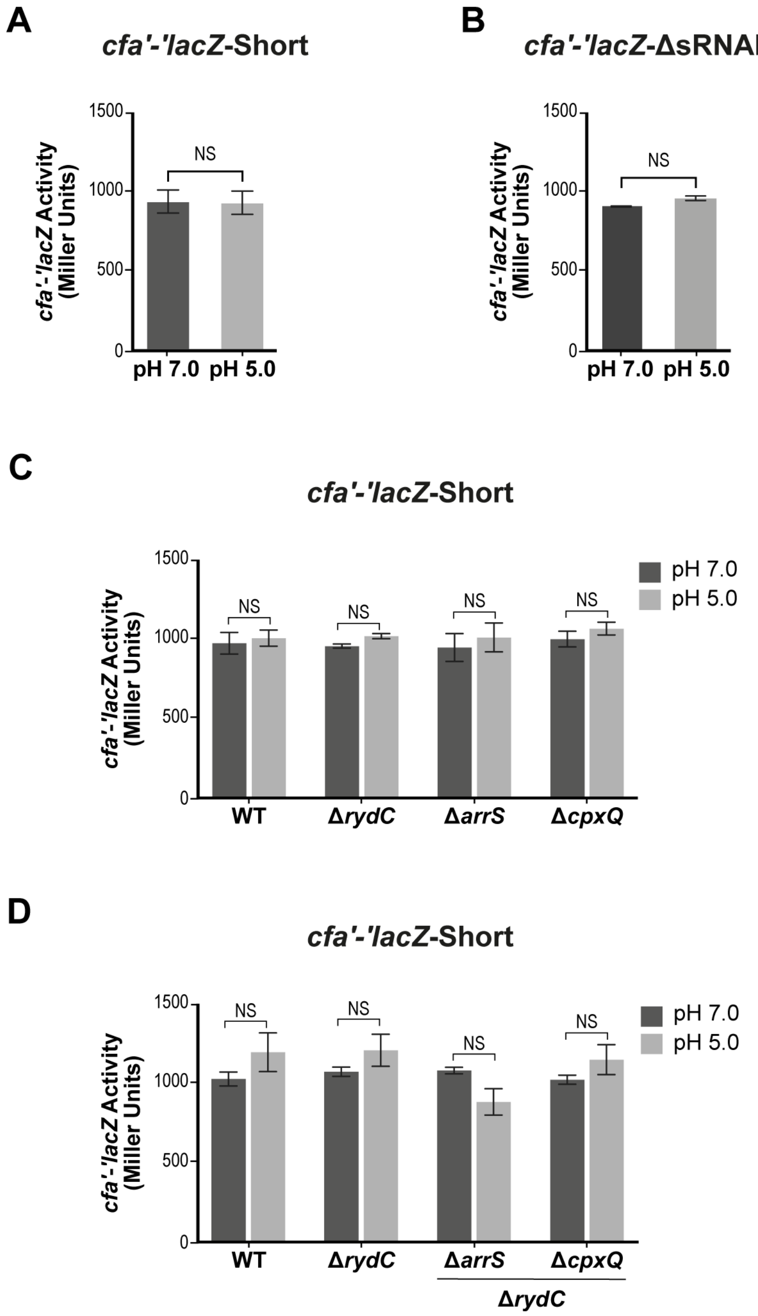

Figure S7: Deletion of sRNAs do not affect *cfa'-'lacZ*-Short activity

(A) Cells carrying *cfa'-'lacZ*-Short were grown and *cfa* translation in response to acid was assayed as described in Fig. 8. The error bars are standard deviations of three biological replicates and the statistical significance was determined using a two-tailed Student's *t*−test, ns means not significant.

(B) Cells carrying *cfa'-'lacZ*-ΔsRNABS were grown and *cfa* translation in response to acid was assayed as described in Figs. 8, S7A.

(C) Cells carrying *cfa'-'lacZ*-Short (in either WT background or a background where one sRNA is deleted) were grown as described in Figs. 8, S7A. The error bars are standard deviations of three biological replicates and the statistical significance was determined using a two-tailed Student's *t*−test, ns means not significant.

(D) Cells carrying *cfa'-'lacZ*-Short (in either WT background or a background where *rydC* and one other sRNA are deleted) were grown as described in Figs. 8, S7A. *ΔrydC* single mutant is included for reference. Error bars represent standard deviation for three biological replicates and the statistical significance was determined using a two-tailed Student's *t*−test, ns means not significant.

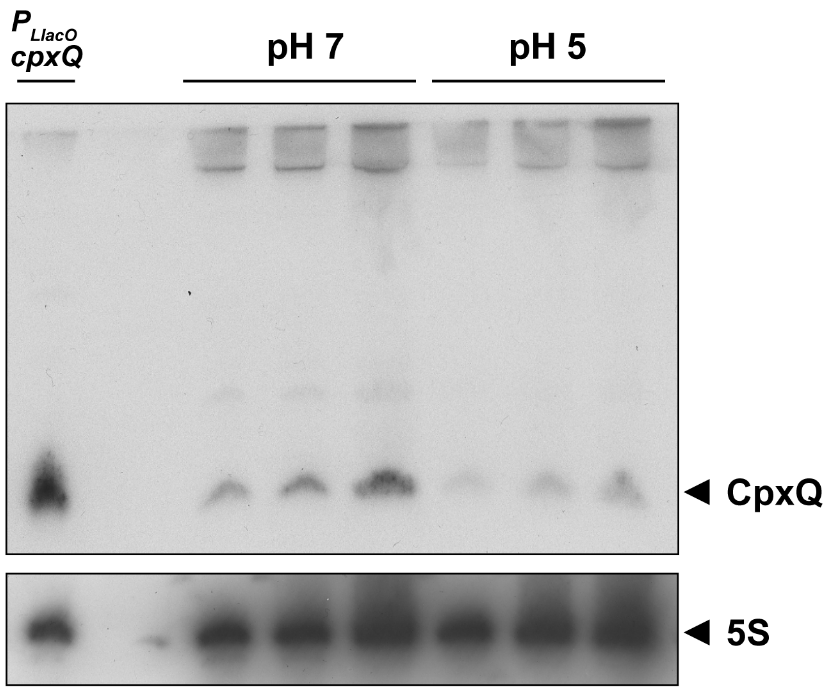

Figure S8: CpxQ expression is higher at pH 7 compared to at pH 5
Expression levels of CpxQ at pH 7 and pH 5 were determined by northern blot analysis of total RNA samples. Wild-type cells were grown in medium at pH 7 then subcultured into medium either pH 7 (three biological replicates) or pH 5 (three biological replicates). Samples were harvested 120 minutes later and RNA was extracted. The P_{LlacO} CpxQ positive control was prepared as described in Fig. 4C. 5S served as loading control.

Table S1: Predicted base-pairing between sRNAs and *cfa* mRNA

IntaRNA was used to predict interactions between each sRNA and *cfa* mRNA. The sRNA (bottom) and *cfa* mRNA (top) are oriented 3′->5′ and 5′->3′, respectively.

<table>
<tr><th>sRNA</th><th>Hybridization free energy (kcal/mol)</th><th>Predicted base-pairing</th></tr>
<tr><td>RydC</td><td>-14.13</td><td>-109 -99
| |
5′ UUCUACGUCGGAU 3′ *cfa*
|||||:|||||
3 CAGAUGUAGCCUU 5′ RydC
| |
12 2</td></tr>
<tr><td>ArrS</td><td>-8.78</td><td>-105 -95
| |
5′ CGUCGGAUUAU 3′ *cfa*
:||||||||:
3′ UUAGCCUAAUG 5′ ArrS
| |
10 1</td></tr>
<tr><td>OxyS</td><td>-3.31</td><td>-188 -182
| |
5′ AAAUAUUAUGUUUUUAC 3′ *cfa*
|:|:|||
3′ UUUCAAGUGCAACCGAA 5′ OxyS
| |
84 78</td></tr>
<tr><td>GadF</td><td>-10.01</td><td>-111 -91
| |
5′ CCTCTGUUCUACGUC-GGAUU-AUAGACTCGCG 3′ *cfa*
||||||| ||||| |:| ||
3′ CTTCCCAAGAUGACCACCUAUGUGUAUGGTCCC 5′ GadF
| |
36 14</td></tr>
<tr><td>GcvB</td><td>-17.45</td><td>-155 -140
| |
5′ CCGACUCACUAUGGAUAGUCAUUUCG 3′ *cfa*
||||||||||:|| ||
3′ UAAUUAGUGAUACCUGUCUGUCCCAU 5′ GcvB
| |
170 155</td></tr>
<tr><td>CpxQ</td><td>-6.99</td><td>-135 -128
| |
5′ AUUUCGGCAAGGGUUC 3′ *cfa*
|||||||:
3′ AGAUACCGUUCCUUUU 5′ CpxQ
| |
11 4</td></tr>
</table>

Table S2: Expression of sRNAs can alter fatty acid composition
Relative qualification of fatty acids in *E. coli* in response to ectopic expression of *arrS*, *cfa*, *cpxQ*, *rydC*, or vector control. Fatty acids are presented as a percent of total identified fatty acids. Error represents average ± standard deviation, n=3 or ^n=2. ND: not detected.

	p_{lac}				
**Fatty Acid**	*arrS*	*cfa^*	*cpxQ*	*rydC^*	*vector*
C12:0	2.5 ± 1.1	1.8 ± 0.7	1.0 ± 0.1	5.2 ± 0.5	1.29 ± 0.08
C14:0	7.4 ± 1.5	6.0 ± 1.0	3.1 ± 0.1	12.6 ± 3.8	3.6 ± 0.4
C14:1	ND	ND	0.18 ± 0.07	ND	ND
C16:0	43.9 ± 1.1	43.3 ± 4.0	30.7 ± 1.6	42.8 ± 0.3	33.6 ± 1.7
C16:0 methyl	0.8 ± 0.6	0.48 ± 0.08	0.8 ± 0.2	ND	0.9 ± 0.2
C16:1	13.5 ± 1.5	10.9 ± 0.7	19.9 ± 1.1	6.2 ± 0.4	18.9 ± 0.7
C17:0 CFA	11.8 ± 1.0	13.7 ± 1.2	1.2 ± 0.3	17.3 ± 3.8	2.2 ± 0.4
C18:0	12.2 ± 1.6	14.4 ± 2.1	25.4 ± 3.6	9.8 ± 2.0	21.7 ±0.6
C18:1	7.9 ± 0.7	9.1 ± 2.0	13.8 ± 1.7	6.0 ±0.7	14.9 ± 0.9
C18:2	ND	ND	ND	ND	ND
C19:0 CFA	ND	0.9 ± 0.3	ND	ND	ND
C20:0	ND	ND	1.1 ± 0.3	ND	0.9 ± 0.1
C22:0	ND	ND	1.1 ± 0.3	ND	0.7 ± 0.2
C24:0	ND	ND	1.1 ± 0.3	ND	0.8 ±0.2
C26:0	ND	ND	0.6 ± 0.3	ND	0.3 ± 0.2

Table S3: Plasmids and strains used in this study

Plasmid	Vector	Genotype	Source or Reference
pBRCS12	pHDB3	vector control	Wadler *et al.*, 2009
pCB1	pBR322	P_{lac}-*5' UTR cfa G101C mutation*	This study
pCB2	pBR322	P_{lac}-*5' UTR cfa U98C, U97C mutation*	This study
pCB3	pBRCS12	P_{lac}-*cfa*	This study
pCB4	pBRCS12	P_{lac}-*arrS*	This study
pCB5	pBRCS12	P_{lac}-*cpxQ*	This study
pCB6	pBRCS12	P_{lac}-*gadF*	This study
pCB7	pBRCS12	P_{lac}-*rydC*	This study
pCB8	pBRCS12	P_{lac}-*arrS M1*	This study
pCB9	pBRCS12	P_{lac}-*rydC M1*	This study
pCB10	pBRCS12	P_{lac}-*cpxQ M2*	This study
pCB11	pBRCS12	P_{lac}-*oxyS*	This study
pCB12	pBRCS12	P_{lac}-*gcvB*	This study
pXG10		vector control	Urban and Vogel, 2007
pKF206	pXG10	P_{LtetO1}-*cfa*	Fröhlich *et al.*, 2013
pBAD		vector control	
pKF523-7	pBAD	P_{BAD}-*cpxQ*	This study
pKF524-1	pBAD	P_{BAD}-*rydC*	This study

Strain	Background	Genotype	Source or Reference
DJ480	MG1655	Δlac X74	D. Jin, NCI*
PM1205	PM1203	lacI':: P_{BAD}-*cat-sacB-lacZ*, miniλtet^{R}, Δ*araBAD araC*+, *mal::lacI^{q}*	Mandin *et al.,* 2009
CB 438	DJ480	λattB::lacIq	This study
CB 533	DJ480	DJ 480 Laq^{IQ} λatt Δ*arrS*	This study
CB 534	DJ480	DJ 480 Laq^{IQ} λatt Δc*pxQ*	This study
CB 535	DJ480	DJ 480 Laq^{IQ} λatt Δg*adF*	This study
CB 536	DJ480	DJ 480 Laq^{IQ} λatt Δr*ydC*	This study
CB 565	DJ480	DJ 480 Laq^{IQ} λatt Δ*cfa*	This study
AK 27	PM1205	P_{BAD}-*cfa'-'lacZ*-Long	King *et al.,* 2019

Table S3 continued

Strain	Background	Genotype	Source or Reference
AK 28	PM1205	P_{BAD}-*cfa'-'lacZ*-Short	King *et al.,* 2019
CB 582	AK 28	P_{BAD}-cfa'-'lacZ-Short Δ*cpxQ*	This study
CB 583	AK 27	P_{BAD} -*cfa'-'lacZ*-Long Δ*cpxQ*	This study
CB 588	AK 27	P_{BAD} -*cfa'-'lacZ*-Long Δ*rydC*	This study
CB 589	AK 28	P_{BAD}-cfa'-'lacZ-Short Δ*rydC*	This study
CB 594	AK 27	P_{BAD} -*cfa'-'lacZ*-Long Δ*arrS*	This study
CB 595	AK 28	P_{BAD}-cfa'-'lacZ-Short Δ*arrS*	This study
CB 600	AK 27	P_{BAD} -*cfa'-'lacZ*-Long Δ*rydC* Δ*cpxQ*	This study
CB 601	AK 28	P_{BAD}-cfa'-'lacZ-Short Δ*rydC* Δ*cpxQ*	This study
CB 611	AK 27	PBAD -cfa'-'lacZ-Long Δ*rydC* Δ*arrS*	This study
CB 612	AK 28	P_{BAD}-cfa'-'lacZ-*Short* Δ*rydC* Δ*arrS*	This study
CB 615	AK 27	P_{BAD} -*cfa'-'lacZ*-Long Δ*rpoS*	This study
CB 622	PM 1205	P_{BAD} -*cfa'-'lacZ*-Long ΔsRNABS	This study
CB 639	PM 1205	P_{BAD} -*cfa'-'lacZ*-Long ΔHfq*BS*	This study
CB 772	PM 1205	P_{BAD} -*cfa'-'lacZ*-LongM1	This study
CB 903	PM 1205	P_{BAD} -*cfa'-'lacZ*-LongM2	This study
CB 812	PM 1205	P_{BAD} -*cfa'-'lacZ*-LongG1	This study
CB 792	PM 1205	P_{BAD} -*cfa'-'lacZ*-LongG2	This study
CB 839	PM 1205	P_{BAD} -*cfa'-'lacZ*-LongG3	This study
CB 840	PM 1205	P_{BAD} -*cfa'-'lacZ*-LongG4	This study
CB 825	PM 1205	P_{BAD} -*cfa'-'lacZ*-LongH1	This study
CB 826	PM 1205	P_{BAD} -*cfa'-'lacZ*-LongH4	This study
CB 980	PM 1205	P_{BAD} -*cfa'-'lacZ*-LongH4G3	This study
*NCI, National Cancer Institute, Frederick, MD			

Table S4: Oligos used in this study

Oligo	Description	Sequence 5'-3'
CB128	Forward primer for FRT-kanR-FRT casette insertion in *arrS*	CCAGTTTGTGATCTCTGAAGAATATTACTAAAGTTAAAATATTCCGGG GATCCGTCGACC
CB131	Reverse primer for FRT-kanR-FRT casette insertion in *arrS*	ACATGAATGCGTTATTTACTCAGGTAATTTCAATGCGTTATGTAGGCT GGAGCTGCTTCG
CB132	Forward primer for FRT-kanR-FRT casette insertion in *rydC*	ATTATGGTTTTATTTATCATACAAATAAATATAATAGGCGATTCCGGGG ATCCGTCGACC
CB133	Reverse primer for FRT-kanR-FRT casette insertion in *rydC*	CTACGCATGATGCCGCGTAAACGTTCCTGAAGGATATTTATGTAGGC TGGAGCTGCTTCG
CB134	Forward primer for FRT-kanR-FRT casette insertion in *gadF*	ATTACCCCCGGTGATTACTAAAGGAGAGGCTAAAACGAATTCCGGG GATCCGTCGACC
CB135	Reverse primer for FRT-kanR-FRT casette insertion in *gadF*	GATACAGGCACAGTGATCGACATGGTGAGGTCAACGACTGTAGGC TGGAGCTGCTTCG
CB136	Forward primer for FRT-kanR-FRT casette insertion in *cpxQ*	GTTGAAGCTATTGAGTAGTAGCAACTCACGTTCCCAGtagATTCCGG GGATCCGTCGACC
CB137	Reverse primer for FRT-kanR-FRT casette insertion in *cpxQ*	GCAAATTGAGGATAAAAAAAACCCCCACAGCATGTGGGGGTGTAG GCTGGAGCTGCTTCG
CB 91	Forward confirmation primer for FRT-kanR-FRT casette cloning in *arrS*	GCGGATTACTGCCCAAGAATAAG
CB 92	Reverse confirmation primer for FRT-kanR-FRT casette cloning in *arrS*	CCGTGTCTCCAGACGCTATATA
CB158	Forward confirmation primer for FRT-kanR-FRT casette cloning in *rydC*	GATACCGTT GATGAGATCA AAGATCGGG
CB159	Reverse confirmation primer for FRT-kanR-FRT casette cloning in *rydC*	CTGCACTGGC ACACCACTAT TTC
CB109	Forward confirmation primer for FRT-kanR-FRT casette cloning in *gadF*	AGGACATAAGCAACTGAAATTGATG
CB 110	Reverse confirmation primer for FRT-kanR-FRT casette cloning in *gadF*	CGAGGAGAGATATGAGGGATT
CB 113	Forward confirmation primer for FRT-kanR-FRT casette cloning in *cpxQ*	GCAATGGCAAAAAAGTTCATCG
CB 114	Reverse confirmation primer for FRT-kanR-FRT casette cloning in *cpxQ*	GACCCGGTATACCACCAT
CB 124	Forward primer for cloning *E. coli ArrS* into pBRCS12 contains Bam site	CCCCCCCCGGATCCGTAATCCGATTTAAATATCGAGTCTC
CB 98	Reverse primer for cloning *E. coli ArrS* and *ArrS M1* into pBRCS12 contains HindIII site	CCCCCCCCAAGCTTCGTTATTTACTCAGGTAATTTCAATGCG
CB125	Forward primer for cloning *E. coli RydC* into pBRCS12 contains Bam site	CCCCCCCCGGATCCCTTCCGATGTAGACCCGTATTCTT
CB100	Reverse primer for cloning *E. coli RydC* and *RydCM1* into pBRCS12 contains HindIII site	CCCCCCCCAAGCTTCCGCGTAAACGTTCCTGAAGGATAT
CB126	Forward primer for cloning *E. coli GadF* into pBRCS12 contains Bam site	CCCCCCCCGGATCCCTTTATTCCCCTGGTATGTGTATCC
CB102	Reverse primer for cloning *E. coli GadF* into pBRCS12 contains HindIII site	CCCCCCCCAAGCTTGATCGACATGGTGAGGTCAAC
CB127	Forward primer for cloning *E. coli CpxQ* into pBRCS12 contains Bam site	CCCCCCCCGGATCCTTTTCCTTGCCATAGACACCATCCC
CB106	Reverse primer for cloning *E. coli CpxQ* and *CpxQM2* into pBRCS12 contains HindIII site	CCCCCCCCAAGCTTTGACGCTAGTATAACGGAAGC
AK24F	Forward primer for cloning *cfa* 5'UTR into PM1205 to make PBAD -cfa'-'lacZ-Long	ACCTGACGCTTTTTATCGCAACTCTCTACTGTTTCTCCATGAGTTGT TAAAGCTACGATAAATATTATGTTTTTACGGGGACAGGATCGT
AK24F2	Forward primer for cloning *cfa* 5'UTR into PM1205 to make PBAD -cfa'-'lacZ-Short	ACCTGACGCTTTTTATCGCAACTCTCTACTGTTTCTCCATAAGGTTC TGATCACCGACCAGTGATGGAGAAA
AK24R	Reverse primer for cloning *cfa* 5'UTR into PM1205 to make PBAD -cfa'-'lacZ-Long, Short, ΔsRNABS, ΔHfqBS PBAD -cfa'-'lacZ-Long H1/ H4, PBAD -cfa'-'lacZ-Long G1-G3	TAACGCCAGGGTTTTCCCAGTCACGACGTTGTAAAACGACCGGGG CAGAACCGTTAATGG
CB175	Forward primer for cloning *cfa* 5'UTR into PM1205 to make PBAD -cfa'-'lacZ-Long ΔsRNABS	ACCTGACGCTTTTTATCGCAACTCTCTACTGTTTCTCCATTTCTGCG AGATTTCTCACAA
CB183	Forward primer for cloning *cfa* 5'UTR into PM1205 to make PBAD -cfa'-'lacZ-Long ΔHfqBS	ACCTGACGCTTTTTATCGCAACTCTCTACTGTTTCTCCAT GACTCACTATGGATAGTCAT
CB 213	Forward primer for cloning *cfa* 5'UTR into PM1205 to make PBAD -cfa'-'lacZ-Long H1	ACCTGACGCTTTTTATCGCAACTCTCTACTGTTTCTCCATGAGTTGT TAAAGCTACGATAAATATTATGTTTCTACG
CB 214	Forward primer for cloning *cfa* 5'UTR into PM1205 to make PBAD -cfa'-'lacZ-Long H4	ACCTGACGCTTTTTATCGCAACTCTCTACTGTTTCTCCATGAGTTGT TAAAGCTACGATAAATATTATGCCCCTACG
CB 204	Forward confirmation primer for gibson plamids	AAGCATTTATCAGGGTTATTGTCTCATGAGC

Table S4 continued

Oligo	Description	Sequence 5'-3'
CB 205	Reverse confirmation primer for gibson plamids	AAACAACTGGCGGTATGGATGC
CB 202	Forward primer for cloning *cfa* 5'UTR using Gibson to make PBAD -cfa'-'lacZ-Long M1 and M2	AAAGCCCAAAAAGCGTCTACGCTGTTTTAAGGTTCTGATCACCGAC<br>CAGTGATGGAGAAACTATGAGTTCATCGTGTATAGAAGAAGTCAGT<br>GTACCGGATGACAACTGGTACCGTATCGCCAACGAATTACTTAGCC<br>GTGCCGGTATAGCCATTAACGGTTCTGCCCCGACATCTGTATTAACG<br>AAGCGCTGG
CB 208	Reverse primer for cloning *cfa* 5'UTR using Gibson to make PBAD -cfa'-'lacZ-Long M1	GTAGACGCTTTTTGGGCTTTGTGAGAAATCTCGCAGAAAAAACCGC<br>GAGTCTATAATCGGACGTAGAACAGAGGGAAAGGAGGAACCCTTG<br>CCGAAATGACTATCCATAGTGAGTCGGGAACGATCCTGTCCCCGTA<br>AAAACATAATATTTATCGTAGCTTTAACAACTCGGTTTCTTAGACGTC<br>AGGTGGC
CB G2	Gene block to make PBAD -cfa'-'lacZ-Long M2	CTGACGCTTTTTATCGCAACTCTCTACTGTTTCTCCATGAGTTGTTA<br>AAGCTACGATAAATATTATGTTTTTACGGGGACAGGATCGTTCCCGA<br>CTCACTATGGATAGTCATTTC*C*GCAAGGGTTCCTCCTTTCCCTCTGT<br>TCTACGTCGGATTATAGACTCGCGGTTTTTTCTGCGAGATTTCTCAC<br>AAAGCCCAAAAAGCGTCTACGCTGTTTTAAGGTTCTGATCACCGAC<br>CAGTGATGGAGAAACTATGAGTTCATCGTGTATAGAAGAAGTCAGT<br>GTACCGGATGACAACTGGTACCGTATCGCCAACGAATTACTTAGCC<br>GTGCCGGTATAGCCATTAACGGTTCTGCCCCGGTCGTTTTACAACG<br>TCGTGACTGGGAAAACCCTGGCGTTA
CB 209	QuickChange primer to make PBAD -cfa'-'lacZ-LongG1	AAAAACCGCGAGTCTATACTCCGACGTAGAACAGAGGGAA
CB 210	QuickChange primer to make PBAD -cfa'-'lacZ-LongG1	TTCCCTCTGTTCTACGTCGGAGTATAGACTCGCGGTTTTT
CB 211	QuickChange primer to make PBAD -cfa'-'lacZ-LongG2	AGAAAAAACCGCGAGTCTATCCTCCGACGTAGAACAGAGGGAAAG
CB 212	QuickChange primer to make PBAD -cfa'-'lacZ-LongG2	CTTTCCCTCTGTTCTACGTCGGAGGATAGACTCGCGGTTTTTTCT
CB Q23	QuickChange primer to make PBAD -cfa'-'lacZ-LongG3	AAAAAACCGCGAGTCTACCCTCCGACGTAGAACAG
CB Q24	QuickChange primer to make PBAD -cfa'-'lacZ-LongG3	CTGTTCTACGTCGGAGGGTAGACTCGCGGTTTTTT
CB 229	Forward primer for cloning *E. coli ArrS M1* into pBRCS12 contains Bam site	CCCCCCCCGGATCCGTAATCGGATTTAAATATCGAGTCTC
CB 231	Forward primer for cloning *E. coli RydC M1* into pBRCS12 contains Bam site	CCCCCCCCGGATCCCTTC*G*GATGTAGACCCGTATTCTT
CB 248	Forward check primer for Δ*rppH*	GGAGTATGAAACAATCATTCGTATATAAAGC
CB 249	Reverse check primer for Δ*rppH*	GCTACCTTTTCGACTATTTCGC
CpxQ NB	DNA oligo for CpxQ northern blot probe	GAAGACAGGGATGGTGTCTATG
5S NB	DNA oligo for 5S northern blot probe	GTTTCACTTCTGAGTTCGGCATGGGGTCAGGTGGG
CB 106	Reverse primer for cloning *E. coli CpxQ1 and CpxQ2* into pBRCS12 contains HindIII site	CCCCCCCCAAGCTTTGACGCTAGTATAACGGAAGC
CB 238	Forward primer for cloning *E. coli CpxQ1* into pBRCS12 contains Bam site	CCCCCCCCGGATCCCGCATAGTTACCGCTGCCG
CB 241	Forward primer for cloning *E. coli CpxQ2* into pBRCS12 contains Bam site	CCCCCCCCGGATCCAGCAACTCACGTTCCCAGTAGT
CB 250	Forward primer for cloning *E. coli OxyS* into pBRCS12 contains Bam site	CCCCCCCCGGATCCGAAACGGAGCGGCACCTCTTTTAA
CB 251	Reverse primer for cloning *E. coli OxyS* into pBRCS12 contains HindIII site	CCCCCCCCAAGCTTCGCCGGGCTTTTTTATGGCA
CB 252	Forward primer for cloning *E. coli GcvB* into pBRCS12 contains Bam site	CCCCCCCCGGATCCACTTCCTGAGCCGGAACGAAAA
CB 253	Reverse primer for cloning *E. coli GcvB* into pBRCS12 contains HindIII site	CCCCCCCCAAGCTTGTAATTCGCGATCGCAAGGTAA
CB 259	Forward check primer for Δ*rpoS*	AGGCTTTTGCTTGAATGTTCCG
CB 260	Reverse check primer for Δ*rpoS*	GAAAAGGCCAGCCTCGCTT
CB 1065	Forward primer for cloning *E. coli CpxQ M2* into pBRCS12 contains Bam site	CCCCCCCCGGATCCTTTTCCTTGCGATAGACACCATCCC

Table S4 continued

Oligo	Description	Sequence 5'-3'
CB GH4G3	Gene block to make PBAD -cfa'-'lacZ-LongH4G3	CTGACGCTTTTTATCGCAACTCTCTACTGTTTCTCCATGAGTTGTTA<br>AAGCTACGATAAATATTATGCCCCTACGGGGACAGGATCGTTCCCG<br>ACTCACTATGGATAGTCATTTCGGCAAGGGTTCCTCCTTTCCCTCTG<br>TTCTACGTCGGAGGGTAGACTCGCGGTTTTTTCTGCGAGATTTCTC<br>ACAAAGCCCAAAAAGCGTCTACGCTGTTTTAAGGTTCTGATCACCG<br>ACCAGTGATGGAGAAACTATGAGTTCATCGTGTATAGAAGAAGTCA<br>GTGTACCGGATGACAACTGGTACCGTATCGCCAACGAATTACTTAG<br>CCGTGCCGGTATAGCCATTAACGGTTCTGCCCCGGTCGTTTTACAA<br>CGTCGTGACTGGGAAAACCCTGGCGTTA
KFO-0702	sense oligo for to amplify *E. coli cfa* mRNA (with T7 promoter)	GTTTTTTTTAATACGACTCACTATAGAGTTGTTAAAGCTACGAT
KFO-0703	antisense oligo for to amplify *E. coli cfa* mRNA	CTCGCAGAAAAAACCGC
KFO-0704	sense oligo for to amplify *E. coli* RydC (with T7 promoter)	GTTTTTTTTAATACGACTCACTATAGGTTCCGATGTAGACCCGTA
KFO-0705	antisense oligo for to amplify *E. coli* RydC	AGAAAACGCCTGTACTAA
KFO-0740	sense oligo for to amplify *E. coli* CpxQ (with T7 promoter)	GTTTTTTTTAATACGACTCACTATAGGTTTTCCTTGCCATAGACACC
KFO-0707	antisense oligo for to amplify *E. coli* CpxQ	AAAAAAAACCCCCACAGCATG
KFO-0708	sense oligo for to amplify *E. coli* ArrS (with T7 promoter)	GTTTTTTTTAATACGACTCACTATAGGTAATCCGATTTAAATATCG
KFO-0709	antisense oligo for to amplify *E. coli* ArrS	AAAGAAAGCTGGCAATCC
KFO-0710	sense oligo for to amplify *E. coli* GadF (with T7 promoter)	GTTTTTTTTAATACGACTCACTATAGGCTTTATTCCCCTGGTATG
KFO-0711	antisense oligo for to amplify *E. coli* GadF	AAAAAAAATGTTGCCGTTCT
KFO-0880	sense oligo for to amplify *E. coli* GcvB (with T7 promoter)	GTTTTTTTTTTAATACGACTCACTATAGGACTTCCTGAGCCGGAACG
KFO-0881	antisense oligo for to amplify *E. coli* GcvB	AAAAAAAGCACCGCAATTAGGC
KFO-0884	sense oligo for to amplify *E. coli* OxyS (with T7 promoter)	GTTTTTTTTTTAATACGACTCACTATAGGAAACGGAGCGGCACCT
KFO-0885	antisense oligo for to amplify *E. coli* OxyS	CAAAAAAAAGCGGATCCTGG
KFO-0826	antisense to *gfp*; primer extension and DNA template *cfa* mRNA sequencing ladder	CCGTATGTAGCATCACCTTC
pZE-Cat	DNA template *cfa* mRNA sequencing ladder	TGGGATATATCAACGGTGGT
KFO-0818	Forward primer for cloning *E. coli RydC* into pBAD	TTCCGATGTAGACCCGTAT
KFO-0819	Reverse primer for cloning *E. coli RydC* into pBAD; with XbaI site	GTTTTTTCTAGAATCGGGTTGCACGCT
KFO-0820	Reverse primer for cloning *E. coli CpxQ1* into pBAD; with XbaI site	GTTTTTTCTAGAGACGCTAGTATAACGGAAGC
JVO-9567	Forward primer for cloning *E. coli CpxQ1* into pBAD; with 5' phosphorylation	P~TTTTCCTTGCCATAGACACC
KFO-0971	antisense oligo H1 to block Hfq binding site	GAACGATCCTGTCCCCGTAAAAACATAAT
